# Charting the evolutionary path of the SUMO modification system in plants reveals molecular hardwiring of development to stress adaptation

**DOI:** 10.1093/plcell/koae192

**Published:** 2024-06-26

**Authors:** Srayan Ghosh, Macarena Mellado Sanchez, Kawinnat Sue-Ob, Dipan Roy, Andrew Jones, Miguel A Blazquez, Ari Sadanandom

**Affiliations:** Department of Biosciences, Durham University, Durham, DH1 3LE, UK; Instituto de Biología Molecular y Celular de Plantas (CSIC-UPV), Valencia, 46022, Spain; Institute of Systems, Molecular and Integrative Biology, University of Liverpool, Liverpool, L69 3BX, UK; Department of Biosciences, Durham University, Durham, DH1 3LE, UK; Institute of Systems, Molecular and Integrative Biology, University of Liverpool, Liverpool, L69 3BX, UK; Instituto de Biología Molecular y Celular de Plantas (CSIC-UPV), Valencia, 46022, Spain; Department of Biosciences, Durham University, Durham, DH1 3LE, UK

## Abstract

SUMO modification is part of the spectrum of Ubiquitin-like (UBL) systems that give rise to proteoform complexity through post-translational modifications (PTMs). Proteoforms are essential modifiers of cell signaling for plant adaptation to changing environments. Exploration of the evolutionary emergence of Ubiquitin-like (UBL) systems unveils their origin from prokaryotes, where it is linked to the mechanisms that enable sulfur uptake into biomolecules. We explore the emergence of the SUMO machinery across the plant lineage from single-cell to land plants. We reveal the evolutionary point at which plants acquired the ability to form SUMO chains through the emergence of SUMO E4 ligases, hinting at its role in facilitating multicellularity. Additionally, we explore the possible mechanism for the neofunctionalization of SUMO proteases through the fusion of conserved catalytic domains with divergent sequences. We highlight the pivotal role of SUMO proteases in plant development and adaptation, offering new insights into target specificity mechanisms of SUMO modification during plant evolution. Correlating the emergence of adaptive traits in the plant lineage with established experimental evidence for SUMO in developmental processes, we propose that SUMO modification has evolved to link developmental processes to adaptive functions in land plants.

## Teaser

The post-translational modification SUMO has evolved to link developmental processes to adaptive functions in land plants.

## Introduction

Variation at the protein level (proteoforms) plays a key role in orchestrating biological complexity (Kosova et al. 2021). Post-translational modification (PTM) events create distinct proteoforms and modulate almost every biological process. This is particularly evident in multicellular organisms, where development requires the integration of signals controlling and coordinating cell fates (switching from division to differentiation). PTMs act at the core of every cellular decision. For example, progression through the eukaryotic cell cycle is regulated by the dynamic interplay of kinases and phosphatases that regulate phosphorylation events controlling a wide range of protein functions from subcellular location to enzyme activity (Ardito et al. 2017). There are >100 different PTMs that modulate every cellular process from transcription to protein stability and function (Vu et al. 2018; Perrar et al. 2019; Kosova et al. 2021).

**Figure 1.**
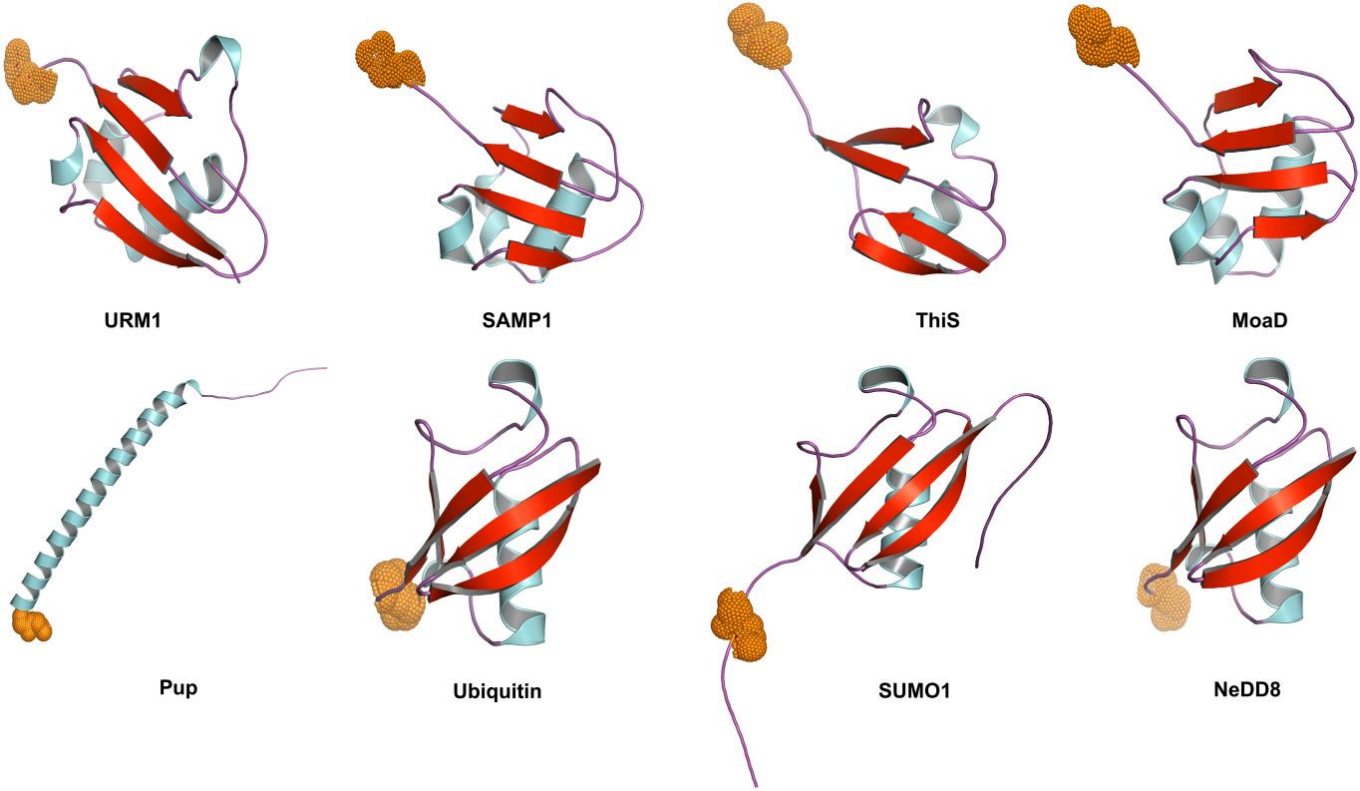
Protein conjugation system in prokaryotes and eukaryotes. The protein modifiers composed of typical ßßαßßß tertiary structures common across all protein conjugation systems. Structure of Ubiquitin-related modifier-1 (URM1) protein from *Saccharomyces cerevisiae*, Small Archaeal Modifier Protein-1 (SAMP1) structure from *Haloferax gibbonsii*, ThiaminS (ThiS) structure in *Escherichia coli* K12, and Molybdenum cofactor biosynthesis protein D (MoaD) structure in *E. coli* K12. However, the analogous peptide modifier, Pup, in *Mycobacterium* sp. (strain KMS) lacks the ßßαßßß tertiary structure. In unicellular algae through land plants, this common ßßαßßß tertiary structure occurs in Ubiquitin, SUMO1, and Nedd8/Rub1.

**Figure 2.**
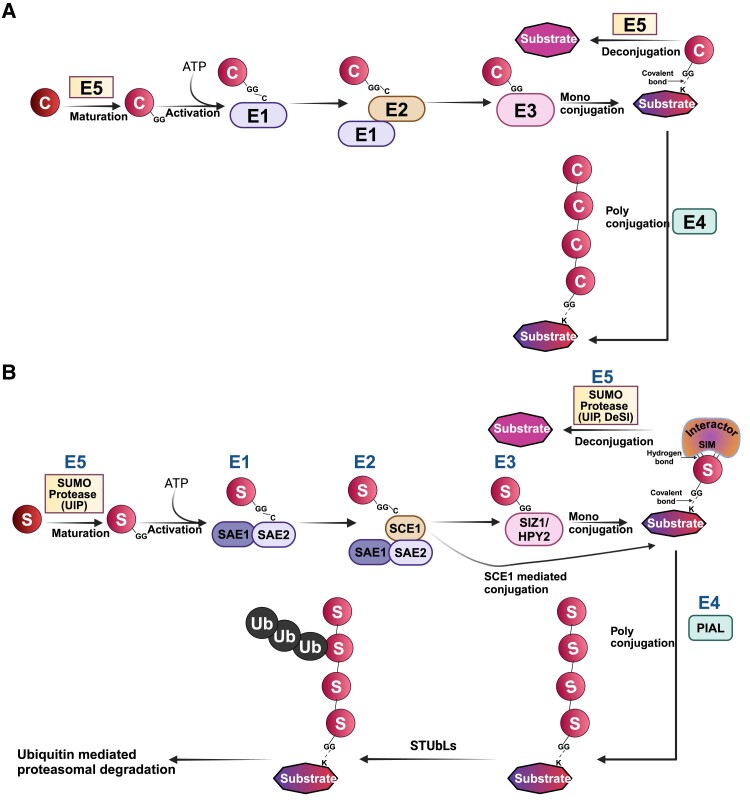
Overview of peptide modification systems. **A)** In general, a battery of enzymes (E1, E2, E3, E4, ES) processes and conjugates the peptide modifier (C) to the protein substrate. The process begins with the modifier being C terminally processed by ES proteases exposing a terminal glycine, G residue and accepted by an ATP-driven enzyme complex composed of E1 and E2 proteins. Subsequently the modifier is transferred to a third enzyme, E3, or the E2 enzyme itself conjugates the modifier to the substrate protein at the Lys residue (K), forming an isopeptide bond. Another class of enzyme known as E4 may be present that links several modifier residues to the substrate (polyconjugation). Additionally, ES proteases help in cleaving off the modifier from the substrate. Ubiquitylation, Neddylation, and SUMOylation are examples of this type of peptide conjugation systems in eukaryotes. **B)** SUMO modification machinery involves a pre-SUMO matured to expose the C-terminal GG motif by Ulp type of SUMO proteases before being linked to a Cys residue of E1 (composed of SAE1 and SAE2 as a heterodimer). This is followed by transfer to a Cys residue on E2 (SCE1) and finally to the E3 ligase SIZ1/HPY2. The E2 or E3 can covalently link SUMO to the Lys residue of the substrate protein. The SUMO E4 can attach polySUMOylation chains to the substrate protein that may eventually mark the protein for ubiquitylation by STUbL. SUMO is cleaved from the substrate protein by the activity of SUMO proteases (both Ulp and DeSI type proteases). The SUMOylated substrate can interact with proteins containing SIMs (SUMO interacting motifs). Abbreviations: DeSI, DeSumoylating lsopeptidase; FUG, Fourth ULP Gene class; HPY, High Ploidy2; OTS, Overly Tolerant to Salt; PIAL, Protein Inhibitor of Activated STAT Like1; SAE, SUMO Activating Enzyme; SCE, SUMO Conjugating Enzyme; SIZ, SAP and MIZ1 domain- containing ligase1; SPF, SUMO Protease related to Fertility; UlP, Ubiquitin-like Proteases.

The ubiquitylation system is one of the first protein conjugation systems to be discovered (Miura and Hasegawa 2010; Callis 2014; Linden and Callis 2020). Ubiquitin, a 76–amino acid protein, is linked via its glycine to lysine residues of substrate proteins through an isopeptide bond (Fig. 1A). The ubiquitin conjugation system—a cascading collection of the enzymes sequentially named E1, E2, E3, and E4—enables this modification. By attaching ubiquitin molecules, proteins can be marked for degradation, localized to specific cellular compartments, or modulated in their activity and interactions (Callis 2014). The ubiquitylation system is involved in fundamental cellular processes, including cell cycle regulation, DNA repair, and protein quality control. In both the plant and animal kingdoms, a large repertoire of proteins has been found to be conjugated to ubiquitin. Further studies into the Ubiquitin system gave rise to the discovery of several other Ubiquitin-like (Ubl) systems with similar conjugating machinery. SUMOylation, NEDDylation, UFMylation, and ISGylation are some of the prominent Ubl conjugation systems (Fig. 1;Fig. 2A) (Hochstrasser 2009; Vierstra 2012; Mergner and Schwechheimer 2014; Villarroya-Beltri et al. 2017; Li et al. 2023).

SUMOylation acting through lysine residues on target proteins (summarized in Fig. 2B) has been firmly established as a vital PTM that affects almost every cellular process studied so far (Benlloch and Lois 2018; Morrell and Sadanandom 2019), but the pivotal importance and regulation of the SUMO system in plants is just beginning to be discovered (Vierstra 2012; Augustine and Vierstra 2018; Benlloch and Lois 2018; Morrell and Sadanandom 2019). SUMOylation involves the attachment of Small Ubiquitin-like Modifier (SUMO) proteins to target proteins, mediated by SUMO conjugation machinery comprising SUMO E1 activating enzyme, SUMO E2 conjugating enzyme, and SUMO E3 ligase. Additionally, SUMO proteases process premature SUMO and remove SUMO from substrates, regulating various developmental and cellular processes (Fig. 2B). SUMO was first discovered as a conjugated protein on Ran-GTPase activating protein1, RanGAP1 in mammals (Matunis et al. 1996). Subsequent studies have reported the presence of a SUMO modification machinery enabling this post-translational modification on target proteins in unicellular yeast (Hochstrasser 2009). The SUMO modification system has been extensively studied in animals, including humans, where SUMOylation is established as a major modifier of cell signaling, particularly during stress responses (Yeh 2009; Bettermann et al. 2012; Flotho and Melchior 2013; Celen and Sahin 2020).

In plants, SUMO modification is emerging as a key mechanism by which complex biological processes from stress perception to chromatin changes are orchestrated at the cellular level (Novatchkova et al. 2012; Vu et al. 2018; Morrell and Sadanandom 2019; Celen and Sahin 2020). SUMO conjugation has been shown to be promoted by a plethora of abiotic and biotic stresses with overlapping and distinct phenotypic outputs depending on target substrates in plants (Novatchkova et al. 2012; Vu et al. 2018; Morrell and Sadanandom 2019). In Arabidopsis, SUMO conjugation predominantly involves 2 different forms: *At*SUMO1 and 2. Mutants that fail to promote SUMO1/2 attachment onto target proteins display deregulated immunity and a drastic inability to cope with abiotic stresses. Recently, we and others have shown that major developmental decision processes are enabled by the SUMO system (Han et al. 2016; Gou et al. 2017; Orosa et al. 2018; Srivastava et al. 2020, 2022; Verma et al. 2021). These data underline the importance of SUMOylation in diverse processes that govern plant development and adaptation to their environment. However, there is a gap in our understanding of the evolution and functional diversification of the SUMO system across the plant kingdom.

In this review, we highlight the major differences and similarities between the machineries that drive ubiquitylation and SUMOylation in plants. We explore the emergence of the SUMO machinery across the plant lineage from single cell to land plants. This has allowed us to identify potentially critical components of the SUMO system that were selected for gene expansion as plants successfully adapted to diverse environmental conditions on land. Correlating the emergence of adaptive traits in the plant lineage with established experimental evidence for SUMO in developmental processes, we propose that SUMO modification has evolved to link developmental processes to adaptive functions in land plants.

## Analogy between SUMOylation and ubiquitylation and key differences

Ubiquitylation, discovered as one of the initial protein conjugation systems, laid the groundwork for understanding post-translational modifications that regulate protein function across the biological spectrum. This discovery was followed by the identification of the SUMO conjugation machinery, marking another significant advance in our comprehension of the cellular regulation of proteoforms (Matunis et al. 1996; Hochstrasser 2009; Celen and Sahin 2020). Most of the foundational research has focused on ubiquitylation, uncovering its novel mechanisms of action and regulatory roles in both the plant and animal kingdoms. Despite these advances in understanding ubiquitylation, the exploration of SUMOylation, particularly within the plant kingdom, remains in its early stages.

Protein conjugation machineries of ubiquitylation and SUMOylation share a common feature: they both require an E1–E2–E3 conjugation cascade. Ubl protein conjugation machinery has been reported in prokaryotes, which involves the attachment of sulfur compounds ThiS (ThiaminS) and MoaD (Molybdenum cofactor biosynthesis protein D) to proteins. This system consists of an E1-like enzyme that attaches sulfur moieties to the C-terminal residues of proteins, forming thiocarboxylates. ThiS and MoaD have the characteristic β-grasp motif (Humbard et al. 2010; Maupin-Furlow 2013, 2014). The presence of a protein conjugation system based on a β-grasp motif-containing protein in prokaryotes suggests the Ubl modifier system could have evolved from simpler sulfur conjugation mechanisms found in prokaryotic predecessors, shaped by the sulfur-rich prebiotic environment.

Perhaps the most fascinating difference between Ubiquitylation and SUMOylation lies in the conjugation machinery of these modification systems. The Ubiquitin gene is encoded as polyubiquitin moieties and occurs as identical tandem repeats in the genome. These chains can attach to the substrate protein via polyubiquitylation, or C-terminal hydrolases process them into single units for conjugation to substrates. Contrastingly, SUMO is encoded as individual units with a C-terminal extension beyond the di-glycine motif, which undergoes proteolytic cleavage, leaving a di-glycine motif to conjugate through the E1–E2–E3 complex.

The mature SUMO or Ub protein is bound to E1 in a high-energy–driven process. The E1-activated ubiquitin/SUMO protein is subsequently transferred to an E2 conjugation enzyme, which interacts with E3 ligases to be conjugated to target substrate lysine residues (Fig. 2A). Finally, the conjugation of ubiquitin/SUMO through isopeptide formation to the substrate occurs through complex formation with the E3 ligase core (Fig. 2A). SUMO/ubiquitin can be cleaved from its substrate proteins via isopeptidase activity of cysteine proteases. These proteases maintain a pool of free SUMO/ubiquitin in the cell (Sadanandom et al. 2012; Yates et al. 2016; Morrell and Sadanandom 2019). Multiple SUMO monomers can join in a tandem manner to form a polySUMO chain on a substrate by a group of E4 ligases known as PROTEIN INHIBITOR OF ACTIVATED STAT LIKE1 (PIAL1) and PIAL2 (Tomanov et al. 2014; Han et al. 2016). This is analogous to Ubiquitin E4 ligases that facilitate Ubiquitin chain formation.

Like Ubiquitin, Nedd8, and ISG proteins, SUMO also has a tertiary structure consisting of the β-grasp orientation (ββαβββ structure) (Fig. 1). SUMO modifiers are encoded by a small gene family in genomes. In Arabidopsis, 8 homologs of SUMO have been identified so far. Among these, SUMO1/2 are the most similar and are analogous to human SUMO2/3 and found to be the most involved in SUMO conjugation followed by SUMO3 (analogous to SUMO1 in humans). The functional importance of SUMO1/2 can be attributed to the fact that the *sumo1sumo2* double mutant in Arabidopsis is embryo lethal. It is worth noting that each homolog of SUMO has a unique sequence at its C-terminal extension, unlike in Ubiquitin. It is expected that this divergence in C-terminal extension in SUMOs in Arabidopsis may impart selectivity for different adaptative functions.

A protein substrate can either undergo SUMOylation or interact through its SUMO Interacting Motif (SIM) with a SUMO-modified target to generate an array of differential proteoforms giving rise to a wide range of protein functionalities. The presence of multiple homologs of SUMO modifiers suggests specificity in regulating different developmental processes. The majority of SUMOylated proteins can form noncovalent interactions with proteins possessing SIMs. The SIM motif is characterized by hydrophobic residues surrounded by acidic amino acids that form a beta-sheet secondary structure (Elrouby et al. 2013). Ubiquitin can interact with Ubiquitin Interacting Motifs (UIM) on proteins (Miller et al. 2004; Gao et al. 2021); however, the presence of UIMs in proteins is not well studied in plants.

The E1 conjugating enzyme in SUMOylation is composed of a heteromeric subunit of SAE (SUMO Activating Enzyme) comprising SAE1 (regulatory) and SAE2 (catalytic) subunits, while for ubiquitin the E1 is constituted by a single E1 protein, UAE (Ubiquitin Active enzyme).

In Arabidopsis alone, there are 37 E2 conjugating enzymes that transfer Ubiquitin to E3 Ubiquitin Ligases or catalyze the transfer of Ubiquitin directly to their substrates (Miricescu et al. 2018). However, only 1 SUMO E2 enzyme (SUMO Conjugating Enzyme 1; SCE1) has been reported so far, which facilitates SUMO conjugation to its substrate protein or to the SUMO E3 Ligase for conjugation onto substrates. SCE1 like SAE2 is critical for the survival of the plant as its deletion is lethal (Saracco et al. 2007).

Ubiquitin E3 ligases are diverse and come in at least 1,500 different forms in Arabidopsis alone. They include HECT, RING, Kelch-type, and U-box proteins, suggesting the convergent evolution of different protein families to perform the same function of facilitating the ubiquitylation of target proteins (Sadanandom et al. 2012). Most of the plant hormone receptors are ubiquitin E3 ligases, highlighting the importance of the Ub systems in plants (Blázquez et al. 2020). On the other hand, the SUMO system to date has only 2 confirmed types of E3 ligases, SIZ1 (SAP and MIZ1 domain-containing ligase1) and MMS21/HPY2 (from here on referred to as HPY2), that facilitate the attachment of SUMO chains to substrate proteins (Gou et al. 2017). HPY2 (High Ploidy2) SUMO E3 ligases are dispensable in Arabidopsis as *siz1hpy2* double mutant plants are viable but have severely reduced growth and development (Castro et al. 2018a). In certain instances, like in yeast and animals, the E2 enzyme SCE1 alone can conjugate SUMO to its substrate protein in plants (Varejao et al. 2020; Ghimire et al. 2021). The contrasting evolutionary paths of ubiquitin and SUMO ligases underscore a fundamental regulatory divergence between the 2 systems: Ubiquitin E3 ligases have evolved through positive selection, diversifying to enable dynamic regulation of ubiquitylation, whereas SUMO E3 ligases have been conserved due to negative selection, maintaining a stable regulatory role.

## SUMOylation in the context of plant terrestrialization

Over the years, researchers have made significant strides in understanding the role of SUMO modification in the responses of a handful of model plants to environmental cues. However, 2 important interconnected questions remain largely unexplored: the relevance of SUMO for crop domestication and plant breeding, and the role of SUMO in the generation of adaptive traits during evolution.

Reconstruction of the evolutionary history of a particular pathway requires the comparative analysis of key extant lineages. Thus, to investigate the participation of SUMO in the transition of plants from an aquatic to the terrestrial environment that occurred almost 500 MYA (Umen 2014; de Vries and Archibald 2018), existing species within key lineages of algae and land plants over this period were studied. Land plants (or embryophytes) are comprised of 2 major lineages: Bryophytes or nonvascular land plants (including mosses, liverworts, and hornworts), and Tracheophytes or vascular plants (including lycophytes, ferns, gymnosperms, and angiosperms). The last common ancestor of land plants was derived from a streptophytic algae, which also gave rise to the sister clade of zygnematophytic algae. The analysis of the streptophytic alga genome has provided critical evidence for the evolution of terrestrialization of plant species (Wodniok et al. 2011; Wang et al. 2020). A study in unicellular photosynthetic chlorophytic alga *Chlamydomonas reinhardtii* (Cr) has provided indications of the minimum SUMO modification system that could be available before plant terrestrialization. *Chlamydomonas*, like budding yeast, contains just 1 copy of each SAE1 and SAE2 component of the SUMO-activating enzyme and 1 copy of SIZ1 and HPY2 SUMO E3 ligases. However, in contrast to budding yeast, *Chlamydomonas* contains 3 homologs of SUMO modifiers, 3 homologs of SCE1, and 12 homologs of SUMO proteases.

Cr has 6 SUMO modifiers out of which only 3 are homologous to Arabidopsis SUMO1-SUMO2. The 161-bp physical separation between 2 of these SUMO modifiers suggests tandem duplications have given rise to these SUMO homologs in the Cr genome (Wang et al. 2008).

Among the SUMO proteases, 6 have the C48 peptidase domain found in ULP-type SUMO proteases, whereas 6 members belong to a DESI group of C97 peptidase domain of SUMO proteases (Lin et al. 2020). In the transition of *C. reinhardtii* cells from their optimal growth temperature of 25°C to stress-inducing 37°C, an upsurge in SUMO-conjugated proteins was observed. Intriguingly a 30-minute treatment resulted in a higher abundance of SUMO-conjugated proteins compared with a 1-h treatment, suggesting that deSUMOylation is a critical factor affecting SUMO conjugate accumulation, perhaps explaining the need for an expanded repertoire of SUMO proteases in Cr. Moreover, SUMOylation was also demonstrated to be critical for facilitating phototrophic movement in *Chlamydomonas* (Wang et al. 2008), providing a glimpse of an ancient role for SUMO in red and blue light signaling akin to what has been identified in Arabidopsis (Sadanandom et al. 2015; Srivastava et al. 2022). *Chlamydomonas* may provide a simpler system to understand how SUMOylation was recruited for light perception and signaling in phototrophic organisms.

Very little is known about SUMO and its targets in primitive land plants, i.e. Tracheophytes. The progression of organismal complexity, as they began to colonize land, particularly in terms of growth form, is understood to be gradual. This evolutionary journey is evident from the simple unicellular structure observed in algae, such as *Mesostigma*, through to filamentous growth patterns observed in *Klebsormidium*, and culminating in more complex multicellular structures found in *Chara*, which include specialized structures resembling rhizoids and stems (Umen 2014). Across this diverse range of species, a remarkable conservation of gene number encoding the core SUMO conjugation machinery (E1–E3) is observed. This evidence suggests that SUMO conjugation is indispensable but not necessarily the driving force behind this evolutionary journey. However, as complexity in form arises in the plant lineage, we observe the appearance and gene expansion of specific components of SUMO modification t act beyond the initial SUMO conjugation step. We explore the evolutionary significance of this selection and how it might have contributed to the emergence of land plants.

## Evolution of the SUMO system in plants

**Figure 3.**
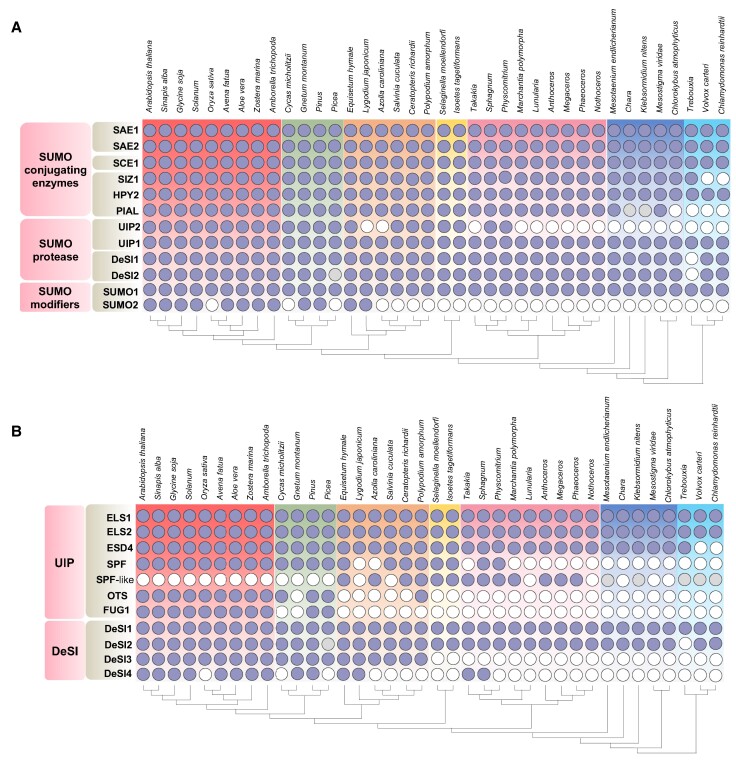
Occurrence of core genes encoding SUMO system genes **(A)** and the expanded SUMO proteases, and **(B)** across different plant lineages. Dark filled circles indicate at least 1 positive hit in a search for the given gene encoding the respective component (rows) in different species (columns), confirmed by phylogenetic analysis. Light-grey filled circles indicate the presence of a plausible or distant ortholog hit. Names of the components are the ones used in Arabidopsis. Different background colors mark the major plant lineages.

To elucidate the evolutionary trajectory of SUMO components throughout the plant kingdom, we conducted an in-depth analysis of sequence homology across several major orders spanning diverse plant lineages. The phylogenetic analysis conducted using publicly available full-genome sequences and data from the OneKP database (One Thousand Plant Transcriptomes 2019) reveals the widespread distribution of almost all components of the SUMO machinery across various plant lineages (Fig. 3A; Supplementary Fig. S2). The occurrence of machinery driving SUMO modifications spans the breadth of the plant kingdom, from unicellular photosynthetic algae to dicots and monocots. Across this spectrum, a core set of SUMO components emerges consistently, including the SUMO modifier (SUMO1), activating enzymes (SAE1 and SAE2), conjugating enzyme (SCE1), ligating enzyme (E3), and proteases (ULP1 and DESI1).

**Figure 4.**
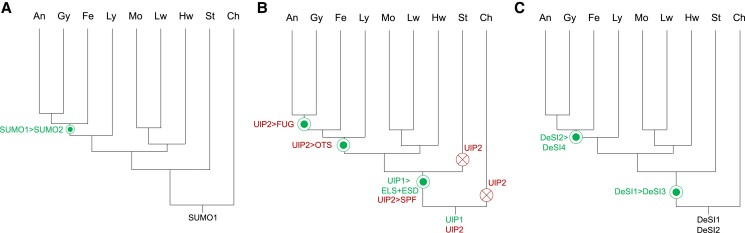
Evolution of SUMO modifiers and proteases in plants. Putative evolutionary trajectory of **(A)** SUMO modifiers, **(B)** Ulp type SUMO proteases, and **(C)** DeSI proteases. Sign “>” indicates the emergence of new SUMO components from the last common ancestor. Green circles indicate the origin of new sub-families by duplication of pre-existing genes. Red crosses indicate putative gene losses in specific lineages. Abbreviations: An, angiosperms; Ch, chlorophytic algae; Fe, ferns; Gy, gymnosperms; Hw, hornworts; Lw, liverworts; Ly, lycophytes; Mo, moses; St, streptophytic algae.

The SUMO1-type proteins most homologous to AtSUMO1 are present in all major lineages while those most homologous to AtSUMO2 are found in fern, Gymnosperms, and Angiosperms, suggesting a more recent emergence (Fig. 4A). It was previously suggested that SUMO3-8 have emerged independently in Brassicaceae (Hammoudi et al. 2016). Arabidopsis SUMO3 had emerged from SUMO2. AtSUMO6 and AtSUMO8 are tandem duplications from AtSUMO4 and AtSUMO7, respectively (Hammoudi et al. 2016). SUMO gene duplication also independently occurs in many other plant species (the same phenomenon being observed in animal SUMO modifiers). It appears that SUMO modifiers have undergone spontaneous gene duplication events across different lineages through the evolution of land plants. This is particularly evident in the cluster of SUMO1-type modifiers where gene duplication has occurred mostly in Bryophytes and Lycophytes, whereas SUMO2-type duplications have evolved mostly in Gymnosperms and Angiosperms. For example, there are 4 copies of SUMO1 homologs in moss *S. fallax* and lycophyte *S. moellendorfii* but 5 and 3 copies on SUMO2 homologs in *P. taeda* and *G. soja*, respectively. Interestingly, the C-terminal end of SUMO, where the processing of the GG motif converts it to its active form, bears considerable diversity across the plant kingdom (Supplementary Fig. S1). Interspecific diversity can be observed in *Sphagnum recurvatum*, *S. lescurii*, and *S. palustre*, which have a free GG motif (or no C-terminal extension) except for *S. fallax* and *S. cuculata*. This suggests that certain species have evolved tighter regulation of SUMOylation by the addition of an extra processing step. However, in *Equisetum hymale* and *Pinus taeda*, we find multiple homologs of SUMO, with and without extensions beyond terminal di-glycine. Interestingly, a variant version of the SUMO modifier also known as SUMO-v was reported in *Zea mays* to have an extensive N-terminal domain preceding the ß-grasp motif but lacked a typical C-terminal GG tail. Genes encoding for SUMO-v were found to be conserved across land plants (Augustine et al. 2016). Another set of tandem di-peptide repeats of ß-grasp motifs known as the DSUL protein was found to be expressed in floral tissues of selected monocot cereal crop species like *Zea mays*, *Brachypodium*, *Panicum*, *Sorghum*, and *Oryza sativa* and also showed a lack of conservation of the GG motif (Augustine et al. 2016). This new finding of 2 different types of SUMO C termini within the same cell type suggests that 2 types of SUMO modification can occur: a fast “hardwired” SUMOylation response when the GG extension is absent or a controlled process through C-terminal processing SUMO proteases as an adaptive response to environmental cues. The presence of an extra copy of SUMO in plants may impart a tissue-specific and/or condition-specific tailored SUMOylation response to different biotic and abiotic stresses. These different modes of interaction with target proteins provide the SUMO system with greater flexibility for modifying cell signaling. The presence of extra copies of SUMO in plants may also impart a tissue-specific and/or condition-specific SUMOylation response to different biotic and abiotic stresses.

The SUMO Activating Enzyme 1 (SAE1) subunit of SUMO E1 activates SUMO to transfer to the E2 enzyme in a 2-step process involving adenylation and thioester bond formation to a catalytic cysteine residue (Lois and Lima 2005). In Arabidopsis alone 2 homologs of SAE1a and SAE1b are found that share 82% homology with each other (Supplementary Fig. S3). The vicinal Asp coordinates Mg^2+^ ion in the ATP-Mg complex and plays an important role in adenylation activity (Lois and Lima 2005; Olsen et al. 2010). SUMO E1s mostly occur as single copy genes, and the catalytic domain is well conserved across the plant kingdom (Supplementary Fig. S4). Interestingly in some algal species, including *Chlamydomonas*, *Mesotaenium*, *Klebsormidium*, and *Penium*, we observed the absence of sequence conservation in either of the Asp or Cys residues. This suggests that photosynthetic algae possess an ancestral form of E1 enzyme that may have a different mechanism to form thiol ester linkage with SUMO proteins. Deleting the SUMO E1 is lethal in eukaryotes, and the acquisition of a catalytic Asp/Cys dyad may be an evolutionary advantage in the terrestrial ecosystem.

The SAE2 (SUMO activating Enzyme 2-catalytic subunit) contains 3 domains comprising of a cysteine rich E1-UbL (Ubiquitin like) domain (C-XX-C) that attaches to a zinc ion, followed by an catalytic cysteine site that forms a E1-SUMO thioester bond and a C-terminal UbL domain consisting of second C-XX-C motif (Lois and Lima 2005). Sequence alignment reveals SAE2 to be a part of a multigene family with several homologs present in a single organism (Supplementary Figs. S5 and S6). However, in several cases homologs across different genera in the plant kingdom show considerable sequence mismatch, particularly at their catalytic sites. This mismatch is commonly observed in algae (*Chlamydomonas*, *Chloromonas*, *Mesotaenium*, *Chara*), bryophytes (*Marchantia*, *Sphagnum*, *Nothoceros*), pteridophytes (*Selaginella*), and some modern angiosperms (*Solanum*, *Glycine*). The predicted SAE2 subunit sequences show limited conservation in 3 of its catalytic domains, which are important for its E1 activity. The appearance of variant isoforms across different plant species indicates that SAE2 has undergone evolutionary divergence, potentially to acquire new functions.

The SUMO Conjugating Enzyme (SCE1) forms a thioester bond with SUMO1 before transferring it to the E3 ligase or the protein substrate (Yunus and Lima 2006). SCE1 is present mostly as a multicopy gene with a highly conserved catalytic active cysteine residue throughout the plant kingdom (Supplementary Figs. S7 and S8; Table 1).

**Table 1.** List of SUMO components including their gene number across different lineages within the plant kingdom

Plant Group	Species	Conjugation components									Proteases									
		SUMO1	SUMO2	SUMO3	SAE1	SAE2	SCE1	SIZ1	HPY2	PIAL	ESD4	ELS1	ELS2	SPF	OTS	FUG1	DESI1	DESI2	DESI3	DESI4
Algae																				
Chlorophyta	*Chlamydomonas reinhardtii*	3		1	1	1	1		1								1	1		
	*Trebouxia arboricola*	2	1		3	3	2	1				3		2						
	*Volvox carteri*	1			1	1	1	1	1								1	1		
Streptophyta	*Chlorokybus atmophyticus*		1		4	3	3	3	1			1		2			1	1		
	*Chara vulgaris*	3		1	1	4	2	1	1								1	1		
	*Klebsormidium subtile*	2	1	1	2	2	4	1	3		1	1	1	1		1	1	1	1	
	*Mesotaenium endlicherianum*	1		1	4	5	3	4	1	3	1	1	1	5			1	1	1	
Bryophyta																				
Liverwort	*Marchantia polymorpha*	1			1	1	1	1	2	1		1					1	1		2
Hornwort	*Nothoceros aenigmaticus*			1	4	3	1	3	2	2	2			4			1	1		
Mosses	*Physchomitrium patens*	2			1	2	2	4	4	1		2				3	2	3		2
	*Sphagnum fallax*	4			1	3	1	5	1	1	1						3	5	4	1
Pteridophyta																				
Lycophyta	*Equisetum hyemale*	3			3	4	10	2	3	2	2	1	2	5	1	1	2	2	4	1
	*Ceratopteris richardii*	2	1		2	2	4	2	1	1	1						3	2	2	1
	*Selaginella moellendorffii*	2			1	1	1	1	2	1		1					2	1		1
	*Isoetes tagetiformans*	2	3	2	3	4	5	8	3	2	1	1	3	2	4	2	1	1	2	1
	*Lygodium japonicum*	2		1	6	4	6	3	3			2	2	1	1		1	1	2	1
	*Polypodium amorphum*	1		1	5	2	7	2	9	2	2		2	2	1		2	2	2	1
	*Polypodium glycyrrhiza*	1		2	5	2	8	3	7	2	2			1	4		2	2	2	1
	*Azolla caroliniana*			1	7	6	9	7	3	2	2	1	2	3	1		1	1	2	
Spermatophyta																				
Gymnosperm	*Cycas micholitzii*	4	1		2	4	3	6	4	1	1	1	2				1	3	3	1
	*Gnetum montanum*	2			3	4	4	5	3	4		1		2	2		1	1	2	1
	*Picea engelmannii*	3	1	3	2	5	3	11	4	4	1		1	1	2	2	2	1	3	
	*Pinus radiata*	4	1	2	4	4		7	8	6		2		1	2	3	2	1	2	1
	*Thuja plicata*	1			1	2	1		1	2		1	1	1	2	1	1	1		2
Angiosperm																				
Diocot	*Amborella trichopoda*	1			1	1	1	1	1	1	1			1	1	1	1	1	2	2
	*Nymphea colorata*	2			1	1	2	1	1	1	1			1	1		1	1	2	2
	*Chenopidium quinoa*	6		2	2	2	2	1	2	2	1		1	2		7	2	2	8	2
	*Glycine soja*	4	1		2	2	4	4	1	4			4	5	2	1	2	3	8	5
	*Populus trichocarpa*	4			2	2	4	2	1	1			2	4	1	2	1	2	9	2
	*Salix purpurea*	4			3	2	4	2	1	2	1		1	3	1	3	1	2	8	3
	*Solanum lycopersicum*	5			2	1	5	2	1	2	1		1	2	2	2	2	2	5	1
	*Brassica oleracea capitata*	0			1	2	4	2	1	3	1			3	1		4	2	8	3
	*Arabidopsis thaliana*	1	1	1	1	2	1	1	1	2	1	1	1	2	2	1	1	1	3	2
	*Citrus sinensis*	5			5	4	5	8	3	4	3		3	5	7	4	1	5	6	3
Monocot	*Yucca filamentosa*	5	1		2	3	3	3	2	5	2		1	2	4	1	2	4	5	4
	*Zostera marina*	1			1	3	1	1	1	3			1	1	2	1	1	1	2	1
	*Eleusine coracana*	3			2	4	7	2	1	1	1		4	2	3	2	4	5	2	4
	*Oryza sativa*	3	1		1	2	2	1	1	1	1		2	1	2	1	2	3	1	3
	*Triticum aestivum*	6	2		6	4	4	6	2	6	3		2	2	4	3	5	6	3	7
	*Brachipodium distachyon*	3			1	1	2	1	2	1	1		2	1	3	3	2	2	1	3
	*Panicum virgatum*	4			3	4	5	3	2	2	2		3	2	8	2	4	5	2	6
	*Zea mays*	2			1	3	4	1	1	1	2		2		3	1	2	2	3	4
	*Sorghum bicolor*	1	1		1	2	3	1	1	1			3	1	5	1	2	3	2	3

The SIZ1 SUMO E3 protein facilitates the transfer of the SUMO molecule from E2 to the lysine residue of the protein substrate. SIZ1 contains a SP-RING domain that forms a tetrahedral configuration of cysteine, histidine, and 2 cysteine molecules coordinating Zn^2+^, which are crucial for its activity (Yunus and Lima 2009). The catalytic sites that contain these amino acids are conserved across the plant kingdom, as shown by the multiple sequence alignment in Supplementary Fig. S9. The SUMO E3 Ligase, HPY2, exhibits conservation from chlorophytes to spermatophytes, whereas SIZ1 is notably absent in *Chlamydomonas*. However, in algae, such as *Spirogloea* and *Coccomyxa*, SIZ1do not exhibit sequence conservation at these catalytic residues, suggesting the existence of an ancestral form of SIZ1 in these early photosynthetic microorganisms. Interestingly, another SUMO E3 ligase, also known as HPY2, is less abundant across the plant kingdom (Supplementary Fig. S10). The sequence alignment shows conservation of cysteine and histidine residues at the catalytic tetrahedral domain. Nevertheless, we observe sequence diversity at these residues in *Chlamydomonas*, *Ostreococcus*, and *Spirogloea* algal species (Supplementary Fig. S11) like SIZ1. Interestingly, SIZ1 and HPY2 always exist as a pair across the plant lineage, which suggests independent parallel evolution in plants as they are functionally not interchangeable. The conserved nature of SUMO E3 ligases and lack of homologs suggest that SIZ1 and HPY2 have specific role in temporal and spatial regulation of SUMO conjugation to protein substrates.

## Evolution of PIAL SUMO E4 ligases

One of the major events in the SUMO system that marked the change from unicellular to multicellular plants was the emergence of SUMO E4 ligases. Arabidopsis encodes 2 SUMO E4 ligases called PROTEIN INHIBITOR OF ACTIVATED STAT LIKE1 (PIAL1) and PIAL2. These SUMO E4 ligases contain SP-RING domains, suggesting that they could also act as E3s. However, they have been demonstrated to create SUMO chains through isopeptide linkages and need SCE1 for its function (Tomanov et al. 2014). Arabidopsis mutant analysis shows that PIAL1 and 2 are required for salt and osmotic stress responses and can alter sulfur metabolism, yet the mutants grow normally under ordinary conditions (Tomanov et al. 2014). PIALs are involved in addition of polySUMOylation chains to the protein substrate, which are bound by STUBL (SUMO-Targeted Ubiquitin Ligases) proteins to be marked for proteasomal degradation (Tomanov et al. 2014; Han et al. 2016). Other than its E4 Ligase activity, PIALs are also involved in transcriptional silencing complex through its interaction with Morpheus’ Molecule 1 (MOM1) containing complex (Han et al. 2016).

In our phylogenetic analysis, we observed that the PIAL proteins were absent in the unicellular algal species. This is corroborated by previous reports showing lack of PIAL proteins in *C. reinhardtii* (Lin et al. 2020). These algal species like *Chloromonas*, *Trebouxia*, and *Chlamydomonas* survive as an independent single-celled organism in the environment (Fig. 2A, Supplementary Figs. S12 and S13). However, with the emergence of multicellular algal species like *Mesotaenium* and *Spirogloea*, where the transition from unicellular to filamentous multicellular structures occurs, we find the emergence of PIALs, which imparts polySUMOylation of protein substrates perhaps to be followed by its subsequent degradation through STUBLs. The SUMO E4 ligase shows sporadic occurrence in organisms belonging to the chlorophytes and streptophytes, emerging more prominently in nonvascular bryophytes (Table 1). Intriguingly, SUMO-conjugated with SCE1 can form SUMO chains even in the absence of PIALs although less efficiently than along with PIAL (Tomanov et al. 2014).

The lack of PIALs in chlorophytic algae suggests that mono and/or multi SUMOylation is the main form of SUMO modification in these unicellular algae. It is tempting to speculate that ability to form PolySUMO chains may be a key feature of the ability to attain multicellularity in the plant lineage (Fig. 3). However, we cannot deny lack of polySUMOylation in these algal species as SIZ1 or HPY2 E3 ligase may also add polySUMO chains to its protein substrate. Further studies need to be undertaken to verify the occurrence of polySUMOylation in unicellular algal species.

## Evolution of SUMO proteases: providing clues for specificity in adaptation

SUMO proteases are cysteine proteases that play a major role in the deconjugation of SUMO from protein substrates. This process helps in the recycling of SUMO components back into the conjugation system and controls cellular SUMO conjugation levels. SUMO proteases also facilitate cleavage of the C-terminal extensions and affect mature SUMO flow into the SUMOylation cycle.

Comparative genomics and phylogenetic analyses have shown that different land plant species have a wide range of SUMO proteases, which shows how important they are for cellular processes (Morrell and Sadanandom 2019). These studies have revealed intriguing patterns of gene duplication, loss, and functional diversification, highlighting the dynamic nature of SUMO proteases in land plant evolution. Additionally, the identification of conserved domains and motifs within these proteases has provided insights into their structural and functional characteristics. Based on the amino acids present in the active site, SUMO proteases can be classified into 2 types. The first is the Ce (Cysteine endopeptidase) class, which mainly consists of ULP (Ubiquitin-Like Proteases) that belong to the C48 protease family. These proteases have a catalytic triad at their active site, which consists of histidine, glutamine (or asparagine), and cysteine (Supplementary Fig. S14). The second type is the CP class of enzymes, which comprises the C97 cysteine protease family. The DeSI (DeSumoylating Isopeptidase) is in this category of proteases. The CP (Cysteine protease) class of enzymes has a characteristic catalytic dyad at its active site, which consists of histidine and cysteine only (Supplementary Fig. S14). The DeSI proteases lack pre-SUMO processing peptidase activity to give rise to mature SUMO forms (Gillies and Hochstrasser 2012; Suh et al. 2012).

The phylogenetic analysis of the ULPs suggests that they can be broadly categorized into 4 groups (Supplementary Fig. S14). ELS (ESD4-Like SUMO protease) and ESD4 (Early in Short Days 4; ULP1-like) and SPF (SUMO Protease related to Fertility; ULP2-like) are present in all lineages, suggesting these are ancient types of ULPs. FUG type ULP proteases are a small group found only in 2 lineages—Gymnosperm and Angiosperm—suggesting that this group has emerged more recently (Castro et al. 2018a). The OTS-type is absent in microalgae, bryophytes, and lycophytes (Fig. 2, B and D). This highlights the neofunctionalization of FUG-type and OTS-type SUMO proteases in more complex plants. Interestingly, we identified another group of ULP2-like proteases related to the SPF-type cluster (we designate as SPF-like), which is exclusively present in bryophytes, lycophytes, and ferns (Fig. 3B, Supplementary S14). This group of proteases indicates the occurrence of a novel, independent parallel evolution of the ULP clade in early non–seed-bearing land plants.

ULP1 is among the first SUMO proteases discovered and was found to regulate the G2/M phase transition in yeast cell cycle (Li and Hochstrasser 1999). In Arabidopsis alone, 8 homologs of ULPs have been reported to be actively involved in modulating a range of stress factors and influencing developmental processes in plants (Morrell and Sadanandom 2019). ESD4 (EARLY IN SHORT DAYS 4) and ELS1 (ESD4 Like SUMO protease) are ULP1 like proteases that have a Ce domain. The catalytic triad consists of histidine, aspartate, and cysteine residues that are conserved across the plant kingdom. It is ubiquitously found in unicellular alga to complex multicellular land plants (Supplementary Fig. S15; Table 1). The critical roles of ULPs in pre-SUMO processing and isopeptidase activity for SUMO modification enable organisms to fine-tune SUMO regulation. This has led to gene expansion and emergence of multiple homologs, perhaps facilitating SUMO mediated adaptation to a wide range of environmental conditions. (Supplementary Fig. S16).

**Figure 5.**
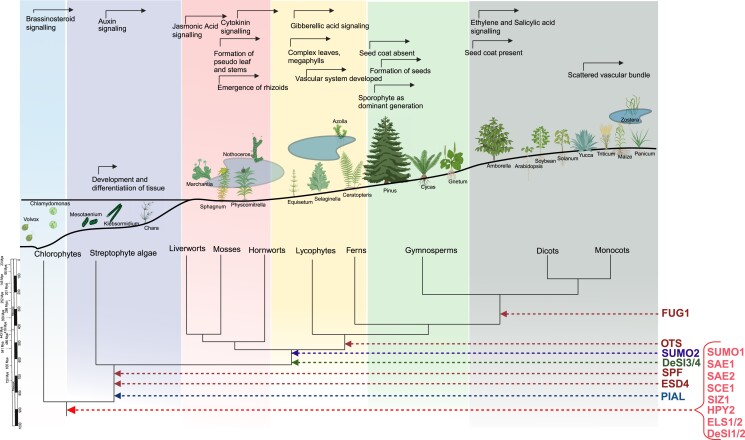
The chronology of the emergence and evolution of the SUMO system components reveal a synchronized progression with the development of various adaptive traits in the plant kingdom. This timeline delineates key evolutionary milestones where specific SUMO components appeared, aligning with the emergence of new functionalities and survival strategies in plants as they adapted to changing environments and ecological niches over millions of years. The cladogram illustrates the timeline indicating the time of the branching points of the main plant lineages.

ELS1/ESD4, ULP type cysteine protease is mostly observed in land plants, indicating a role for adaptation to terrestrial environments. ELS2 and ESD4 appear in streptophytes, while ELS1 is present in chlorophyta and is common in the early vascular and nonvascular plants (Fig. 4B). The evolution of ELS1 in *Chlamydomonas* appears to parallel the development of Brassinosteroid (BR) signaling pathways in chlorophytes. Recent studies in liverworts, showed that the transcription factor BZR1/BES1 levels, regulated by BR signaling, is linked to the facilitation of gametophyte formation (Furuya et al. 2024). ELS1(ULP1a) regulates the SUMOylation status of Arabidopsis BZR1, a major plant transcription factor in the BR pathway. ULP1a aids BZR1 activity in signaling during salt stress to adapt plant growth (Srivastava et al. 2020). Remarkably, we observe a clear correlation between the presence of ELS1/ESD4 ULPs in the plant lineage and the evolutionary emergence of BR signaling, which is thought to have originated in single cell plants (Fig. 5) (Ferreira-Guerra et al. 2020; Kour et al. 2021). Moreover, in response to elevated ambient temperatures, the DESI3a-mediated deSUMOylation of the BR receptor BRI can dampen BR-mediated plant growth (Naranjo-Arcos et al. 2023). These findings underscore the coevolutionary dynamics between SUMO proteases and phytohormones in shaping adaptive responses across plant taxa. By modulating the activity of key signaling components, such as BZR1 and BRI, SUMO proteases intricately regulate plant growth and stress responses in a manner that allows developmental processes to be integrated with environmental cues.

OTS1 and OTS2, known as OVERLY TOLERANT TO SALT 1 and 2, represent a separate category of ULP1 cysteine proteases (Supplementary Fig. S25). They are absent in bryophytes (*Marchantia*, *Physchomitrium*, *Anthoceros*, *Takakia*, and *Sphagnum*), pteridophytes (*Selaginella*, *Equisetum*, *Polypodium*), gymnosperms (*Gnetum*, *Pinus*, *Picea*), and a few angiosperms (*Amborella*, *Solanum*, *Aloe*, *Zostera*) (Supplementary Fig. S26). OTS arises from tracheophytes and undergoes several rounds of endoduplications across spermatophytes. This reveals that OTS SUMO proteases have evolved mostly in angiosperms. The presence of OTS SUMO proteases in angiosperms suggests that these proteases have played a role in the evolution and adaptation of flowering plants. OTS levels play a critical role in controlling plant responses against salt and drought stress in Arabidopsis and rice (Conti et al. 2008; Srivastava et al. 2016a, 2016b). Their absence in algae and bryophytes further highlights their significance in the development of more complex plant structures and functions (Fig. 5). This suggests a multifaceted role for OTS in orchestrating stress adaptation mechanisms in angiosperms. The occurrence of the defense hormone salicylic acid and its signaling mechanism has been elaborated in angiosperms (Monte 2023). It has been reported that OTS1 can downregulate salicylic acid levels to control plant immunity against bacterial pathogens (Bailey et al. 2016). Furthermore, OTS1 levels can also promote SUMOylation of JAZ proteins to attenuate JA signaling (Srivastava et al. 2018). This suggests that the evolution of OTS in angiosperms facilitated the regulation of defense pathways in land plants. Additionally, OTS plays an important role in controlling transcriptional gene silencing by modulating the activity of DNA Polymerase V (Liu et al. 2017a). In addition to its role in stress response, OTS has been found to regulate flowering and stamen and seed development in Arabidopsis (Campanaro et al. 2016; Srivastava et al. 2016b). This implies that the presence of OTS might have played a significant role in shaping the processes of gametophytic generation and seed development in angiosperms (Fig. 5).

The transition to terrestrial life in plants was marked by the evolution of active vascular systems, enabling long-distance nutrient transport and mechanical support (Blázquez et al. 2020; Preston et al. 2022). This adaptation was crucial for the diversification of spermatophytes, with significant variations in root cellular anatomy between angiosperms and gymnosperms (Koonin 2010; de Vries and Archibald 2018; Motte and Beeckman 2019). Lateral root development, originating from the pericycle's stem cell zone or merophyte, exhibits distinct patterns between these groups (Motte and Beeckman 2019). Lycophytes display a rigid merophyte arrangement limiting lateral root formation, whereas the flexible arrangement in angiosperms supports rapid growth and adaptability to soil conditions (Chen et al. 2016). Angiosperm trees, with their larger root diameters, enhanced branching, and efficient xylem vessels, are adapted for better water translocation compared with the tracheid-dominated gymnosperms (Sperry et al. 2006; Liese et al. 2017; Motte and Beeckman 2019). This anatomical difference underpins the angiosperms’ ability to thrive in varied environmental conditions, including colder and nutrient-poor soils, through increased root lignification and nitrogen uptake (Zanne et al. 2014). Conversely, gymnosperms’ lower root proliferation and branching are suited to stable environments with consistent leaf litter (Liese et al. 2017).

The overexpression of OTS1 SUMO protease facilitates increased root length and branching, whereas its silencing reduces root growth (Conti et al. 2008; Srivastava et al. 2016a). The role of OTS1 SUMO protease in promoting root elongation and branching in angiosperms highlights the evolutionary significance of SUMO proteases in adapting root development to terrestrial challenges, demonstrating the intricate link between SUMOylation and plant adaptation to new environments.

SPF1 and SPF2 (SUMO PROTEASE RELATED TO FERTILITY 1/2) belong to the ULP2 class of SUMO proteases having both SUMO maturation and proteolytic activity. They are completely absent in algae but are present in some classes of vascular plants including pteridophytes (Supplementary Fig. S23; Table 1). They occur as a homologous pair SPF1 and SPF2 in Arabidopsis. The catalytic triad residue comprising of histidine, aspartate, and cysteine is conserved throughout (Supplementary Fig. S24). The occurrence of SPF in land plants suggests SPF proteases to have evolved in Tracheophytes playing a role in the evolution of a predominant sporophytic generation giving rise to seed-bearing Spermatophytes (gymnosperms and angiosperms). This evolutionary development may have played a crucial role in the reproductive success and survival of gymnosperms and angiosperms. SPF1 along with SPF2 is important for maintenance of plant fertility and affects gametophyte development as well as embryo formation in Arabidopsis (Liu et al. 2017b; Castro et al. 2018b; Liu et al. 2019). SPF promotes photomorphogenesis in the presence of red light by deSUMOylation of MYC2 whereas this phenomenon is reversed under blue light (Srivastava et al. 2022). The ability of SPF in sensing and responding to different wavelengths of light by altering the SUMOylation status of its substrates highlights the adaptive responses underpinned by SUMO in land plants to convert different environmental cues into developmental signals (Fig. 4).

The ULP2 type protease FUG1 (Fourth ULP Gene class1) has been identified as having emerged relatively recently, tracing back to the Cretaceous period coinciding with the rise of Spermatophytes (Fig. 4). A recent study has elucidated FUG1's role in deSUMOylating the epigenetic gene silencer AL3 (Alfin-like family) induced by repeat expansion induced epigenetic gene silencing, consequently triggering histone methylation (H3K4me) and impeding plant growth by interacting with the Polycomb repressor complex (PRC), a process akin to genomic imprinting (Sureshkumar et al. 2024). In Angiosperms, the development of endosperm and seed formation critically hinges on the genetic imprinting status of seed development genes (Bauer and Fischer 2011; El-Sappah et al. 2021). The emergence of FUG1 in seed-bearing spermatophytes and its involvement in establishing methylation marks for seed development suggests an evolutionary adaptation of SUMO proteases to facilitate the molecular reconfiguration of developmental traits.

The catalytic domain of DeSI1 (comprising of cysteine and histidine) is present in most plants from unicellular algae to land plants (Supplementary Fig. S17). Although DeSI1 has undergone gene expansion, the catalytic residues are conserved throughout its homologs (Supplementary Fig. S18). Interestingly, in *Oryza sativa*, DeSI1 is found to lack a cysteine residue at its catalytic site. This absence suggests the possibility of neofunctionalization occurring in DeSI1, possibly because of selection pressure for high-yielding crops during the process of domestication. Similar to DeSI1, DeSI2 is present as a multigene family and has its catalytic domain is conserved throughout algae, bryophytes and tracheophytes (Supplementary Figs. S19 and S20). Intriguingly in *Polypodium hesperium*, a lycophyte we find a DeSI2 isoform lacking the catalytic cysteine residue. This isoform could potentially be an ancestral gene in ferns from which the present day DeSI2 has arisen. The presence of DeSI1 and DeSI2 homologs in unicellular algae to land plants suggests an important role in photoautotrophic nutrient acquisition (Fig. 3C). DeSI3A, DeSI3C, and DeSI4 also possess cysteine and histidine residues in their catalytic domain (Supplementary Figs. S21, S22 and S27). They are absent in unicellular algae but have arisen as a unigene family in certain multicellular algal species. They are also found in certain bryophytes and pteridophytes. However, they are most commonly found in modern land plants where they have undergone gene expansion (Fig. 5). These findings suggest that DeSI3 and DeSI4 have acquired a role in the evolution and adaptation of plants to terrestrial environments. Notably DeSI3a regulates SUMOylation of FLS2, the bacterial flagellin receptor critical for mounting a potent immune response (Orosa et al. 2018). In this context the occurrence of DeSI3/4 along with its targets across Pteridophytes (such as *Selaginella, Physcomitrella*) can divulge the identity of key immune related processes that have allowed plants to colonize land (Fig. 5). Presently, there is a limited understanding of the potential targets of DeSI in plants, making it a focal point for future understanding.

In summary, the discovery of diverse SUMO components across the plant kingdom, from unicellular algae to multicellular land plants, sheds light on the evolution of SUMO components that responds to adaptability of these organisms to different environmental conditions. In this context neofunctionalization of SUMO proteases as opposed to SUMO E3s within plant genomes highlights a different path taken by SUMO modification when compared withubiquitin as plants adapted to land. The presence or absence of these proteases in various plant lineages provides insights into their role in the adaptation of plants to terrestrial environments and the development of complex structures. The findings presented in this review contribute to unravelling the intricate protein modification mechanisms that govern biological systems, laying the groundwork for future research addressing the role of SUMO and more generally peptide-based modification in early land plants.

## Sequence identification and phylogenetic analysis methods

Sequences of SUMO machinery components in the green lineage were initially gathered with a BlastP local blast search in several databases including OneKP (One Thousand Plant Transcriptomes 2019) Phytozome (Goodstein et al. 2012) and published whole-genome sequences, using an E-value cutoff of 0.1 in most cases. The first search was performed with the corresponding *A. thaliana* sequences, and subsequent searches were done with bryophyte and algal sequences until no new sequences were retrieved. Subsequently, the results were manually checked using SMART (http://smart.emblheidelberg.de/) and Pfam (http://pfam.sanger.ac.uk/search) to ensure the presence of specific domains associated with the different components. A preliminary alignment and tree were performed with the OneClick/FastTree tool available through the NGPhylogeny website (Lemoine et al. 2019) which was then used to discard non-orthologous sequences.

The final alignments were obtained with MAFFT, which were followed by a combination of automatic BMGE (Criscuolo and Gribaldo 2010) and manual inspection to select the optimal region of phylogenetic inference. Phylogenetic trees were built using PhyML with Smart Model Selection (SMS) (Guindon et al. 2010). Bootstrap support was calculated with 1,000 replicates. The graphical representation of the phylogenetic trees was generated using iTOL (Letunic and Bork 2021) and the trees were rooted by using the midpoint. The final figures were edited manually. The models were created using Biorender.

## Supplementary Material

koae192_Supplementary_Data

## Data Availability

The data underlying this article are available in the article and in its online supplementary material.

## Supplementary data

The following materials are available in the online version of this article.

**Supplementary Figure S1.** Multiple sequence alignment of SUMO modifiers highlighting the conservation of GG motif in different species across the plant kingdom.

**Supplementary Figure S2.** Phylogenetic analysis of SUMO modifiers.

**Supplementary Figure S3.** Multiple sequence alignment of SAE1.

**Supplementary Figure S4.** Phylogenetic analysis of SAE1.

**Supplementary Figure S5.** Multiple sequence alignment of SAE2.

**Supplementary Figure S6.** Phylogenetic analysis of SAE2.

**Supplementary Figure S7.** Multiple sequence alignment of SCE1.

**Supplementary Figure S8.** Phylogenetic analysis of SCE1.

**Supplementary Figure S9.** Multiple sequence alignment of SIZ1.

**Supplementary Figure S10.** Phylogenetic analysis of HPY2.

**Supplementary Figure S11.** Multiple sequence alignment of HPY2.

**Supplementary Figure S12.** Phylogenetic analysis of PIAL.

**Supplementary Figure S13.** Multiple sequence alignment of PIAL.

**Supplementary Figure S14.** Phylogenetic analysis of UIP type proteases.

**Supplementary Figure S15.** Phylogenetic analysis of UIP1.

**Supplementary Figure S16.** Multiple sequence alignment of UlP.

**Supplementary Figure S17.** Phylogenetic analysis of DeSI1.

**Supplementary Figure S18.** Multiple sequence alignment of DeSI1.

**Supplementary Figure S19.** Phylogenetic analysis of DeSI2.

**Supplementary Figure S20.** Multiple sequence alignment of DeSI2.

**Supplementary Figure S21.** Phylogenetic analysis of DeSI3.

**Supplementary Figure S22.** Multiple sequence alignment of DeSI3.

**Supplementary Figure S23.** Phylogenetic analysis of SPF.

**Supplementary Figure S24.** Multiple sequence alignment of SPF.

**Supplementary Figure S25.** Phylogenetic analysis of OTS.

**Supplementary Figure S26.** Multiple sequence alignment of OTS.

**Supplementary Figure S27.** Phylogenetic analysis of DeSI4.

**Supplementary Data Sets 1 to 39.** Fasta sequence alignments (*.fasta) and Newick gene tree (*.nhx) files.

## Dive Curated Terms

The following phenotypic, genotypic, and functional terms are of significance to the work described in this paper:

SIZ1 Gramene: AT5G60410
SIZ1 Araport: AT5G60410

SUMO1 Gramene: AT4G26840
SUMO1 Araport: AT4G26840

BZR1 Gramene: AT1G75080
BZR1 Araport: AT1G75080

SIM Gramene: AT5G04470
SIM Araport: AT5G04470

SUMO2 Gramene: AT5G55160
SUMO2 Araport: AT5G55160

SUMO3 Gramene: AT5G55170
SUMO3 Araport: AT5G55170

FUG1 Gramene: AT1G17220
FUG1 Araport: AT1G17220

FLS2 Gramene: AT5G46330
FLS2 Araport: AT5G46330

MYC2 Gramene: AT1G32640
MYC2 Araport: AT1G32640

OTS2 Gramene: AT1G10570
OTS2 Araport: AT1G10570

ESD4 Gramene: AT4G15880
ESD4 Araport: AT4G15880

OTS1 Gramene: AT1G60220
OTS1 Araport: AT1G60220

SAE2 Gramene: AT2G21470
SAE2 Araport: AT2G21470

PIAL1 Gramene: AT1G08910
PIAL1 Araport: AT1G08910

DESI1 Gramene: AT3G07090
DESI1 Araport: AT3G07090

ELS1 Gramene: AT3G06910
ELS1 Araport: AT3G06910

SPF1 Gramene: AT1G09730
SPF1 Araport: AT1G09730

SCE1 Gramene: AT3G57870
SCE1 Araport: AT3G57870

## References

[koae192-B1] Ardito F , Giuliani M, Perrone D, Troiano G, Lo Muzio L. The crucial role of protein phosphorylation in cell signaling and its use as targeted therapy (rReview). Int J Mol Med. 2017:40(2):271–280. doi:10.3892/ijmm.2017.3036 28656226 PMC5500920

[koae192-B2] Augustine RC , Vierstra RD. SUMOylation: re-wiring the plant nucleus during stress and development. Curr Opin Plant Biol. 2018:45(Pt A):143–154. doi:10.1016/j.pbi.2018.06.006 30014889

[koae192-B3] Augustine RC , York SL, Rytz TC, Vierstra RD. Defining the SUMO sSystem in mMaize: SUMOylation is up-regulated during endosperm development and rapidly induced by stress. Plant Physiol. 2016:171(3):2191–2210. doi:10.1104/pp.16.00353 27208252 PMC4936565

[koae192-B4] Bailey M , Srivastava A, Conti L, Nelis S, Zhang C, Florance H, Love A, Milner J, Napier R, Grant M, et al Stability of small ubiquitin-like modifier (SUMO) proteases OVERLY TOLERANT TO SALT1 and -2 modulates salicylic acid signalling and SUMO1/2 conjugation in Arabidopsis thaliana. J Exp Bot. 2016:67(1):353–363. doi:10.1093/jxb/erv468 26494731 PMC4682439

[koae192-B5] Bauer MJ , Fischer RL. Genome demethylation and imprinting in the endosperm. Curr Opin Plant Biol. 2011:14(2):162–167. doi:10.1016/j.pbi.2011.02.006 21435940 PMC3082360

[koae192-B6] Benlloch R , Lois LM. Sumoylation in plants: mechanistic insights and its role in drought stress. J Exp Bot. 2018:69(19):4539–4554. doi:10.1093/jxb/ery233 29931319

[koae192-B7] Bettermann K , Benesch M, Weis S, Haybaeck J. SUMOylation in carcinogenesis. Cancer Lett. 2012:316(2):113–125. doi:10.1016/j.canlet.2011.10.036 22138131

[koae192-B8] Blázquez MA , Nelson DC, Weijers D. Evolution of plant hormone response pathways. Annu Rev Plant Biol. 2020:71(1):327–353. doi:10.1146/annurev-arplant-050718-100309 32017604

[koae192-B9] Callis J . The ubiquitination machinery of the ubiquitin system. Arabidopsis Book. 2014:12:e0174. doi:10.1199/tab.0174 25320573 PMC4196676

[koae192-B10] Campanaro A , Battaglia R, Galbiati M, Sadanandom A, Tonelli C, Conti L. SUMO proteases OTS1 and 2 control filament elongation through a DELLA-dependent mechanism. Plant Reprod. 2016:29(4):287–290. doi:10.1007/s00497-016-0292-8 27761651

[koae192-B11] Castro PH , Bachmair A, Bejarano ER, Coupland G, Lois LM, Sadanandom A, van den Burg HA, Vierstra RD, Azevedo H. Revised nomenclature and functional overview of the ULP gene family of plant deSUMOylating proteases. J Exp Bot. 2018a:69(19):4505–4509. doi:10.1093/jxb/ery301 30124991 PMC6117577

[koae192-B12] Castro PH , Santos MA, Freitas S, Cana-Quijada P, Lourenco T, Rodrigues MAA, Fonseca F, Ruiz-Albert J, Azevedo JE, Tavares RM, et al Arabidopsis thaliana SPF1 and SPF2 are nuclear-located ULP2-like SUMO proteases that act downstream of SIZ1 in plant development. J Exp Bot. 2018b:69(19):4633–4649. doi:10.1093/jxb/ery265 30053161 PMC6117582

[koae192-B13] Celen AB , Sahin U. Sumoylation on its 25th anniversary: mechanisms, pathology, and emerging concepts. FEBS J. 2020:287(15):3110–3140. doi:10.1111/febs.15319 32255256

[koae192-B14] Chen W , Koide RT, Adams TS, DeForest JL, Cheng L, Eissenstat DM. Root morphology and mycorrhizal symbioses together shape nutrient foraging strategies of temperate trees. Proc Natl Acad Sci U S A. 2016:113(31):8741–8746. doi:10.1073/pnas.1601006113 27432986 PMC4978252

[koae192-B15] Conti L , Price G, O'Donnell E, Schwessinger B, Dominy P, Sadanandom A. Small ubiquitin-like modifier proteases OVERLY TOLERANT TO SALT1 and -2 regulate salt stress responses in Arabidopsis. Plant Cell. 2008:20(10):2894–2908. doi:10.1105/tpc.108.058669 18849491 PMC2590731

[koae192-B16] Criscuolo A , Gribaldo S. BMGE (block mMapping and gGathering with eEntropy): a new software for selection of phylogenetic informative regions from multiple sequence alignments. BMC Evol Biol. 2010:10(1):210. doi:10.1186/1471-2148-10-210 20626897 PMC3017758

[koae192-B17] de Vries J , Archibald JM. Plant evolution: landmarks on the path to terrestrial life. New Phytol. 2018:217(4):1428–1434. doi:10.1111/nph.14975 29318635

[koae192-B18] El-Sappah AH , Yan K, Huang Q, Islam MM, Li Q, Wang Y, Khan MS, Zhao X, Mir RR, Li J, et al Comprehensive mechanism of gene silencing and its role in plant growth and development. Front Plant Sci. 2021:12:705249. doi:10.3389/fpls.2021.705249 34589097 PMC8475493

[koae192-B19] Elrouby N , Bonequi MV, Porri A, Coupland G. Identification of Arabidopsis SUMO-interacting proteins that regulate chromatin activity and developmental transitions. Proc Natl Acad Sci U S A. 2013:110(49):19956–19961. doi:10.1073/pnas.1319985110 24255109 PMC3856783

[koae192-B20] Ferreira-Guerra M , Marques-Bueno M, Mora-Garcia S, Cano-Delgado AI. Delving into the evolutionary origin of steroid sensing in plants. Curr Opin Plant Biol. 2020:57:87–95. doi:10.1016/j.pbi.2020.06.005 32861054

[koae192-B21] Flotho A , Melchior F. Sumoylation: a regulatory protein modification in health and disease. Annu Rev Biochem. 2013:82(1):357–385. doi:10.1146/annurev-biochem-061909-093311 23746258

[koae192-B22] Furuya T , Ohashi-Ito K, Kondo Y. Multiple roles of brassinosteroid signaling in vascular development. Plant Cell Physiol. 2024:pcae037. 10.1093/pcp/pcae037 38590039

[koae192-B23] Gao Q , Zhang N, Wang WQ, Shen SY, Bai C, Song XJ. The ubiquitin-interacting motif-type ubiquitin receptor HDR3 interacts with and stabilizes the histone acetyltransferase GW6a to control the grain size in rice. Plant Cell. 2021:33(10):3331–3347. doi:10.1093/plcell/koab194 34323980 PMC8505875

[koae192-B24] Ghimire S , Tang X, Liu W, Fu X, Zhang H, Zhang N, Si H. SUMO conjugating enzyme: a vital player of SUMO pathway in plants. Physiol Mol Biol Plants. 2021:27(10):2421–2431. doi:10.1007/s12298-021-01075-2 34744375 PMC8526628

[koae192-B25] Gillies J , Hochstrasser M. A new class of SUMO proteases. EMBO Rep. 2012:13(4):284–285. doi:10.1038/embor.2012.34 22422001 PMC3321164

[koae192-B26] Goodstein DM , Shu S, Howson R, Neupane R, Hayes RD, Fazo J, Mitros T, Dirks W, Hellsten U, Putnam N, et al Phytozome: a comparative platform for green plant genomics. Nucleic Acids Res. 2012:40(D1):D1178–D1186. doi:10.1093/nar/gkr944 22110026 PMC3245001

[koae192-B27] Gou M , Huang Q, Qian W, Zhang Z, Jia Z, Hua J. Sumoylation E3 ligase SIZ1 modulates plant immunity partly through the immune receptor gene SNC1 in Arabidopsis. Mol Plant Microbe Interact. 2017:30(4):334–342. doi:10.1094/MPMI-02-17-0041-R 28409535

[koae192-B28] Guindon S , Dufayard JF, Lefort V, Anisimova M, Hordijk W, Gascuel O. New algorithms and methods to estimate maximum-likelihood phylogenies: assessing the performance of PhyML 3.0. Syst Biol. 2010:59(3):307–321. doi:10.1093/sysbio/syq010 20525638

[koae192-B29] Hammoudi V , Vlachakis G, Schranz ME, van den Burg HA. Whole-genome duplications followed by tandem duplications drive diversification of the protein modifier SUMO in aAngiosperms. New Phytol. 2016:211(1):172–185. doi:10.1111/nph.13911 26934536 PMC6680281

[koae192-B30] Han YF , Zhao QY, Dang LL, Luo YX, Chen SS, Shao CR, Huang HW, Li YQ, Li L, Cai T, et al The SUMO E3 ligase-like proteins PIAL1 and PIAL2 interact with MOM1 and form a novel complex required for transcriptional silencing. Plant Cell. 2016:28(5):1215–1229. doi:10.1105/tpc.15.00997 27113777 PMC4904672

[koae192-B31] Hochstrasser M . Origin and function of ubiquitin-like proteins. Nature. 2009:458(7237):422–429. doi:10.1038/nature07958 19325621 PMC2819001

[koae192-B32] Humbard MA , Miranda HV, Lim J-M, Krause DJ, Pritz JR, Zhou G, Chen S, Wells L, Maupin-Furlow JA. Ubiquitin-like small archaeal modifier proteins (SAMPs) in Haloferax volcanii. Nature. 2010:463(7277):54–60. doi:10.1038/nature08659 20054389 PMC2872088

[koae192-B33] Koonin EV . The origin and early evolution of eukaryotes in the light of phylogenomics. Genome Biol. 2010:11(5):209. doi:10.1186/gb-2010-11-5-209 20441612 PMC2898073

[koae192-B34] Kosova K , Vitamvas P, Prasil IT, Klima M, Renaut J. Plant proteoforms under environmental stress: functional proteins arising from a single gene. Front Plant Sci. 2021:12:793113. doi:10.3389/fpls.2021.793113 34970290 PMC8712444

[koae192-B35] Kour J , Kohli SK, Khanna K, Bakshi P, Sharma P, Singh AD, Ibrahim M, Devi K, Sharma N, Ohri P, et al Brassinosteroid signaling, crosstalk and, physiological functions in plants under heavy metal stress. Front Plant Sci. 2021:12:608061. doi:10.3389/fpls.2021.608061 33841453 PMC8024700

[koae192-B36] Lemoine F , Correia D, Lefort V, Doppelt-Azeroual O, Mareuil F, Cohen-Boulakia S, Gascuel O. NGPhylogeny.fr: new generation phylogenetic services for non-specialists. Nucleic Acids Res. 2019:47(W1):W260–W265. doi:10.1093/nar/gkz303 31028399 PMC6602494

[koae192-B37] Letunic I , Bork P. Interactive Tree Of Life (iTOL) v5: an online tool for phylogenetic tree display and annotation. Nucleic Acids Res. 2021:49(W1):W293–W296. doi:10.1093/nar/gkab301 33885785 PMC8265157

[koae192-B39] Li B , Niu F, Zeng Y, Tse MK, Deng C, Hong L, Gao S, Lo SW, Cao W, Huang S, et al Ufmylation reconciles salt stress-induced unfolded protein responses via ER-phagy in Arabidopsis. Proc Natl Acad Sci U S A. 2023:120(5):e2208351120. doi:10.1073/pnas.2208351120 36696447 PMC9945950

[koae192-B38] Li SJ , Hochstrasser M. A new protease required for cell-cycle progression in yeast. Nature. 1999:398(6724):246–251. doi:10.1038/18457 10094048

[koae192-B40] Liese R , Alings K, Meier IC. Root branching is a leading root trait of the plant economics spectrum in temperate trees. Front Plant Sci. 2017:8:315. doi:10.3389/fpls.2017.00315 28337213 PMC5340746

[koae192-B41] Lin YL , Chung CL, Huang PJ, Chen CH, Fang SC. Revised annotation and extended characterizations of components of the Chlamydomonas reinhardtii SUMOylation system. Plant Direct. 2020:4(9):e00266. doi:10.1002/pld3.266 33015534 PMC7522501

[koae192-B42] Linden KJ , Callis J. The ubiquitin system affects agronomic plant traits. J Biol Chem. 2020:295(40):13940–13955. doi:10.1074/jbc.REV120.011303 32796036 PMC7535913

[koae192-B43] Liu L , Jiang Y, Zhang X, Wang X, Wang Y, Han Y, Coupland G, Jin JB, Searle I, Fu YF, et al Two SUMO proteases SUMO PROTEASE RELATED TO FERTILITY1 and 2 are required for fertility in Arabidopsis. Plant Physiol. 2017b:175(4):1703–1719. doi:10.1104/pp.17.00021 29066667 PMC5717720

[koae192-B44] Liu L , Yan X, Kong X, Zhao Y, Gong Z, Jin JB, Guo Y. Transcriptional gene silencing maintained by OTS1 SUMO protease requires a DNA-dependent polymerase V-dependent pathway. Plant Physiol. 2017a:173(1):655–667. doi:10.1104/pp.16.01365 27852949 PMC5210737

[koae192-B45] Liu Y , Zhu J, Sun S, Cui F, Han Y, Peng Z, Zhang X, Wan S, Li G. Defining the function of SUMO system in pod development and abiotic stresses in pPeanut. BMC Plant Biol. 2019:19(1):593. doi:10.1186/s12870-019-2136-9 31884953 PMC7194008

[koae192-B46] Lois LM , Lima CD. Structures of the SUMO E1 provide mechanistic insights into SUMO activation and E2 recruitment to E1. EMBO J. 2005:24(3):439–451. doi:10.1038/sj.emboj.7600552 15660128 PMC548657

[koae192-B47] Matunis MJ , Coutavas E, Blobel G. A novel ubiquitin-like modification modulates the partitioning of the Ran-GTPase-activating protein RanGAP1 between the cytosol and the nuclear pore complex. J Cell Biol. 1996:135(6 Pt 1):1457–1470. doi:10.1083/jcb.135.6.1457 8978815 PMC2133973

[koae192-B48] Maupin-Furlow JA . Archaeal pProteasomes and sSampylation. In: Subcellular biochemistry. Dordrecht, The Netherlands: Springer Netherlands; 2013. p. 297–327.

[koae192-B49] Maupin-Furlow JA . Prokaryotic ubiquitin-like protein modification. Ann Rev Microbiol. 2014:68:155–175. doi:10.1146/annurev-micro-091313-103447 24995873 PMC4757901

[koae192-B50] Mergner J , Schwechheimer C. The NEDD8 modification pathway in plants. Front Plant Sci. 2014:5:103. doi:10.3389/fpls.2014.00103 24711811 PMC3968751

[koae192-B51] Miller SL , Malotky E, O'Bryan JP. Analysis of the role of ubiquitin-interacting motifs in ubiquitin binding and ubiquitylation. J Biol Chem. 2004:279(32):33528–33537. doi:10.1074/jbc.M313097200 15155768

[koae192-B52] Miricescu A , Goslin K, Graciet E. Ubiquitylation in plants: signaling hub for the integration of environmental signals. J Exp Bot. 2018:69(19):4511–4527. doi:10.1093/jxb/ery165 29726957

[koae192-B53] Miura K , Hasegawa PM. Sumoylation and other ubiquitin-like post-translational modifications in plants. Trends Cell Biol. 2010:20(4):223–232. doi:10.1016/j.tcb.2010.01.007 20189809

[koae192-B54] Monte I . Jasmonates and salicylic acid: evolution of defense hormones in land plants. Curr Opin Plant Biol. 2023:76:102470. doi:10.1016/j.pbi.2023.102470 37801737

[koae192-B55] Morrell R , Sadanandom A. Dealing with stress: a review of plant SUMO proteases. Front Plant Sci. 2019:10:1122. doi:10.3389/fpls.2019.01122 31620153 PMC6759571

[koae192-B56] Motte H , Beeckman T. The evolution of root branching: increasing the level of plasticity. J Exp Bot. 2019:70(3):785–793. doi:10.1093/jxb/ery409 30481325

[koae192-B57] Naranjo-Arcos M , Srivastava M, Deligne F, Bhagat PK, Mansi M, Sadanandom A, Vert G. SUMO/deSUMOylation of the BRI1 brassinosteroid receptor modulates plant growth responses to temperature. Proc Natl Acad Sci U S A. 2023:120(4):e2217255120. doi:10.1073/pnas.2217255120 36652487 PMC9942830

[koae192-B58] Novatchkova M , Tomanov K, Hofmann K, Stuible HP, Bachmair A. Update on sumoylation: defining core components of the plant SUMO conjugation system by phylogenetic comparison. New Phytol. 2012:195(1):23–31. doi:10.1111/j.1469-8137.2012.04135.x 22799003 PMC3399776

[koae192-B59] Olsen SK , Capili AD, Lu X, Tan DS, Lima CD. Active site remodelling accompanies thioester bond formation in the SUMO E1. Nature. 2010:463(7283):906–912. doi:10.1038/nature08765 20164921 PMC2866016

[koae192-B60] One Thousand Plant Transcriptomes Initiative . One thousand plant transcriptomes and the phylogenomics of green plants. Nature. 2019:574(7780):679–685. doi:10.1038/s41586-019-1693-2 31645766 PMC6872490

[koae192-B61] Orosa B , Yates G, Verma V, Srivastava AK, Srivastava M, Campanaro A, De Vega D, Fernandes A, Zhang C, Lee J, et al SUMO conjugation to the pattern recognition receptor FLS2 triggers intracellular signalling in plant innate immunity. Nat Commun. 2018:9(1):5185. doi:10.1038/s41467-018-07696-8 30518761 PMC6281677

[koae192-B62] Perrar A , Dissmeyer N, Huesgen PF. New beginnings and new ends: methods for large-scale characterization of protein termini and their use in plant biology. J Exp Bot. 2019:70(7):2021–2038. doi:10.1093/jxb/erz104 30838411 PMC6460961

[koae192-B63] Preston JC , Sinha NR, Torii KU, Kellogg EA. Plant structure and function: eEvolutionary origins and underlying mechanisms. Plant Physiol. 2022:190(1):1–4. doi:10.1093/plphys/kiac320 35775936 PMC9434258

[koae192-B64] Sadanandom A , Adam E, Orosa B, Viczian A, Klose C, Zhang C, Josse EM, Kozma-Bognar L, Nagy F. SUMOylation of phytochrome-B negatively regulates light-induced signaling in Arabidopsis thaliana. Proc Natl Acad Sci U S A. 2015:112(35):11108–11113. doi:10.1073/pnas.1415260112 26283376 PMC4568247

[koae192-B65] Sadanandom A , Bailey M, Ewan R, Lee J, Nelis S. The ubiquitin-proteasome system: central modifier of plant signalling. New Phytol. 2012:196(1):13–28. doi:10.1111/j.1469-8137.2012.04266.x 22897362

[koae192-B66] Saracco SA , Miller MJ, Kurepa J, Vierstra RD. Genetic analysis of SUMOylation in Arabidopsis: conjugation of SUMO1 and SUMO2 to nuclear proteins is essential. Plant Physiol. 2007:145(1):119–134. doi:10.1104/pp.107.102285 17644626 PMC1976578

[koae192-B67] Sperry JS , Hacke UG, Pittermann J. Size and function in conifer tracheids and angiosperm vessels. Am J Bot. 2006:93(10):1490–1500. doi:10.3732/ajb.93.10.1490 21642096

[koae192-B68] Srivastava AK , Orosa B, Singh P, Cummins I, Walsh C, Zhang C, Grant M, Roberts MR, Anand GS, Fitches E, et al SUMO suppresses the activity of the jasmonic acid receptor CORONATINE INSENSITIVE1. Plant Cell. 2018:30(9):2099–2115. doi:10.1105/tpc.18.00036 30115737 PMC6181023

[koae192-B71] Srivastava AK , Zhang C, Sadanandom A. Rice OVERLY TOLERANT TO SALT 1 (OTS1) SUMO protease is a positive regulator of seed germination and root development. Plant Signal Behav. 2016a:11(5):e1173301. doi:10.1080/15592324.2016.1173301 27119209 PMC4973764

[koae192-B72] Srivastava AK , Zhang C, Yates G, Bailey M, Brown A, Sadanandom A. SUMO is a critical regulator of salt stress responses in rice. Plant Physiol. 2016b:170(4):2378–2391. doi:10.1104/pp.15.01530 26869703 PMC4825142

[koae192-B69] Srivastava M , Srivastava AK, Orosa-Puente B, Campanaro A, Zhang C, Sadanandom A. SUMO conjugation to BZR1 enables brassinosteroid signaling to integrate environmental cues to shape plant growth. Curr Biol. 2020:30(8):1410–1423.e3. doi:10.1016/j.cub.2020.01.089 32109396 PMC7181186

[koae192-B70] Srivastava M , Srivastava AK, Roy D, Mansi M, Gough C, Bhagat PK, Zhang C, Sadanandom A. The conjugation of SUMO to the transcription factor MYC2 functions in blue light-mediated seedling development in Arabidopsis. Plant Cell. 2022:34(8):2892–2906. doi:10.1093/plcell/koac142 35567527 PMC9338799

[koae192-B73] Suh HY , Kim JH, Woo JS, Ku B, Shin EJ, Yun Y, Oh BH. Crystal structure of DeSI-1, a novel deSUMOylase belonging to a putative isopeptidase superfamily. Proteins. 2012:80(8):2099–2104. doi:10.1002/prot.24093 22498933

[koae192-B74] Sureshkumar S , Bandaranayake C, Lv J, Dent CI, Bhagat PK, Mukherjee S, Sarwade R, Atri C, York HM, Tamizhselvan P, et al SUMO protease FUG1, histone reader AL3 and chromodomain protein LHP1 are integral to repeat expansion-induced gene silencing in Arabidopsis thaliana. Nat Plants. 2024:10(5):749–759. doi:10.1038/s41477-024-01672-5 38641663

[koae192-B75] Tomanov K , Zeschmann A, Hermkes R, Eifler K, Ziba I, Grieco M, Novatchkova M, Hofmann K, Hesse H, Bachmair A. Arabidopsis PIAL1 and 2 promote SUMO chain formation as E4-type SUMO ligases and are involved in stress responses and sulfur metabolism. Plant Cell. 2014:26(11):4547–4560. doi:10.1105/tpc.114.131300 25415977 PMC4277223

[koae192-B76] Umen JG . Green algae and the origins of multicellularity in the plant kingdom. Cold Spring Harb Perspect Biol. 2014:6(11):a016170. doi:10.1101/cshperspect.a016170 25324214 PMC4413236

[koae192-B77] Varejao N , Lascorz J, Li Y, Reverter D. Molecular mechanisms in SUMO conjugation. Biochem Soc Trans. 2020:48(1):123–135. doi:10.1042/BST20190357 31872228

[koae192-B78] Verma V , Srivastava AK, Gough C, Campanaro A, Srivastava M, Morrell R, Joyce J, Bailey M, Zhang C, Krysan PJ, et al SUMO enables substrate selectivity by mitogen-activated protein kinases to regulate immunity in plants. Proc Natl Acad Sci U S A. 2021:118(10):e2021351118. doi:10.1073/pnas.2021351118 33649235 PMC7958252

[koae192-B79] Vierstra RD . The expanding universe of ubiquitin and ubiquitin-like modifiers. Plant Physiol. 2012:160(1):2–14. doi:10.1104/pp.112.200667 22693286 PMC3440198

[koae192-B80] Villarroya-Beltri C , Guerra S, Sanchez-Madrid F. ISGylation— - a key to lock the cell gates for preventing the spread of threats. J Cell Sci. 2017:130(18):2961–2969. doi:10.1242/jcs.205468 28842471

[koae192-B81] Vu LD , Gevaert K, De Smet I. Protein language: post-translational modifications talking to each other. Trends Plant Sci. 2018:23(12):1068–1080. doi:10.1016/j.tplants.2018.09.004 30279071

[koae192-B83] Wang S , Li L, Li H, Sahu SK, Wang H, Xu Y, Xian W, Song B, Liang H, Cheng S, et al Genomes of early-diverging streptophyte algae shed light on plant terrestrialization. Nat Plants. 2020:6(2):95–106. doi:10.1038/s41477-019-0560-3 31844283 PMC7027972

[koae192-B82] Wang Y , Ladunga I, Miller AR, Horken KM, Plucinak T, Weeks DP, Bailey CP. The small ubiquitin-like modifier (SUMO) and SUMO-conjugating system of Chlamydomonas reinhardtii. Genetics. 2008:179(1):177–192. doi:10.1534/genetics.108.089128 18493050 PMC2390597

[koae192-B84] Wodniok S , Brinkmann H, Glockner G, Heidel AJ, Philippe H, Melkonian M, Becker B. Origin of land plants: do conjugating green algae hold the key? BMC Evol Biol. 2011:11(1):104. doi:10.1186/1471-2148-11-104 21501468 PMC3088898

[koae192-B85] Yates G , Srivastava AK, Sadanandom A. SUMO proteases: uncovering the roles of deSUMOylation in plants. J Exp Bot. 2016:67(9):2541–2548. doi:10.1093/jxb/erw092 27012284

[koae192-B86] Yeh ET . SUMOylation and De-SUMOylation: wrestling with life's processes. J Biol Chem. 2009:284(13):8223–8227. doi:10.1074/jbc.R800050200 19008217 PMC2659178

[koae192-B87] Yunus AA , Lima CD. Lysine activation and functional analysis of E2-mediated conjugation in the SUMO pathway. Nat Struct Mol Biol. 2006:13(6):491–499. doi:10.1038/nsmb1104 16732283

[koae192-B88] Yunus AA , Lima CD. Structure of the Siz/PIAS SUMO E3 ligase Siz1 and determinants required for SUMO modification of PCNA. Mol Cell. 2009:35(5):669–682. doi:10.1016/j.molcel.2009.07.013 19748360 PMC2771690

[koae192-B89] Zanne AE , Tank DC, Cornwell WK, Eastman JM, Smith SA, FitzJohn RG, McGlinn DJ, O’Meara BC, Moles AT, Reich PB, et al Three keys to the radiation of angiosperms into freezing environments. Nature. 2014:506(7486):89–92. doi:10.1038/nature12872 24362564

