# Supplementary material for: Charting the evolutionary path of the SUMO modification system in plants reveals molecular hardwiring of development to stress adaptation
Source: Plant Cell. 2024 Jun 26;36(9):3131–44. doi: 10.1093/plcell/koae192 (PMC11371177; doi:10.1093/plcell/koae192)
Supplement: koae192_Supplementary_Data [file koae192_supplementary_data.zip › Supplementary Figures.pdf]

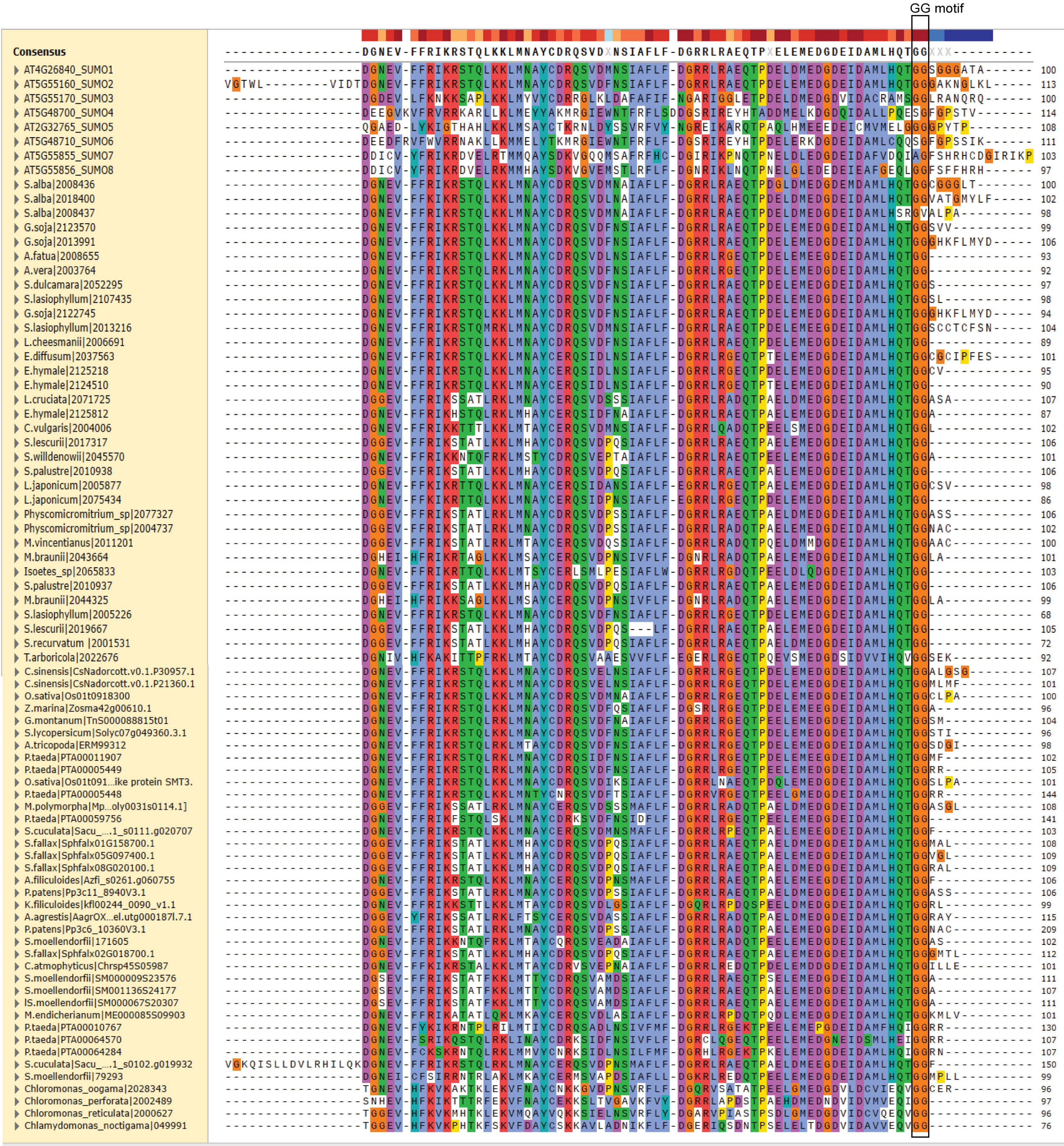

Figure S1: MSA of SUMO modifier highlighting the conservation of GG motif in different species across the plant kingdom

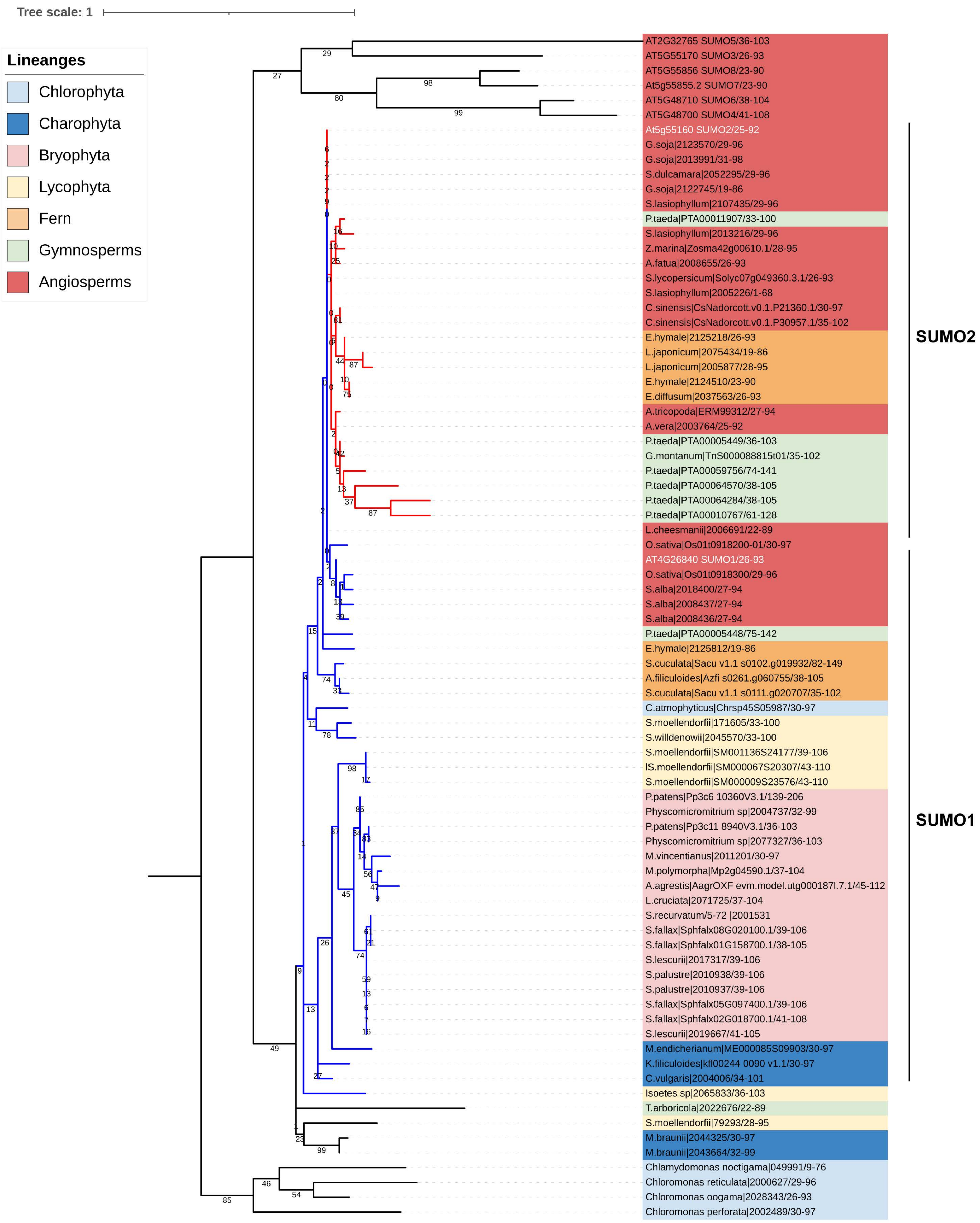

Figure S2: Phylogenetic analysis of SUMO modifiers. The corresponding alignments were obtained and processed as indicated in the text and the tree was constructed using PhyML. Bootstrap support was calculated for 1000 trees, and it is indicated in the branches, with values >0.5 shownb in ticker lines. The tree is drawn to scale, with branch lengths measuring the number of substitutions per site.

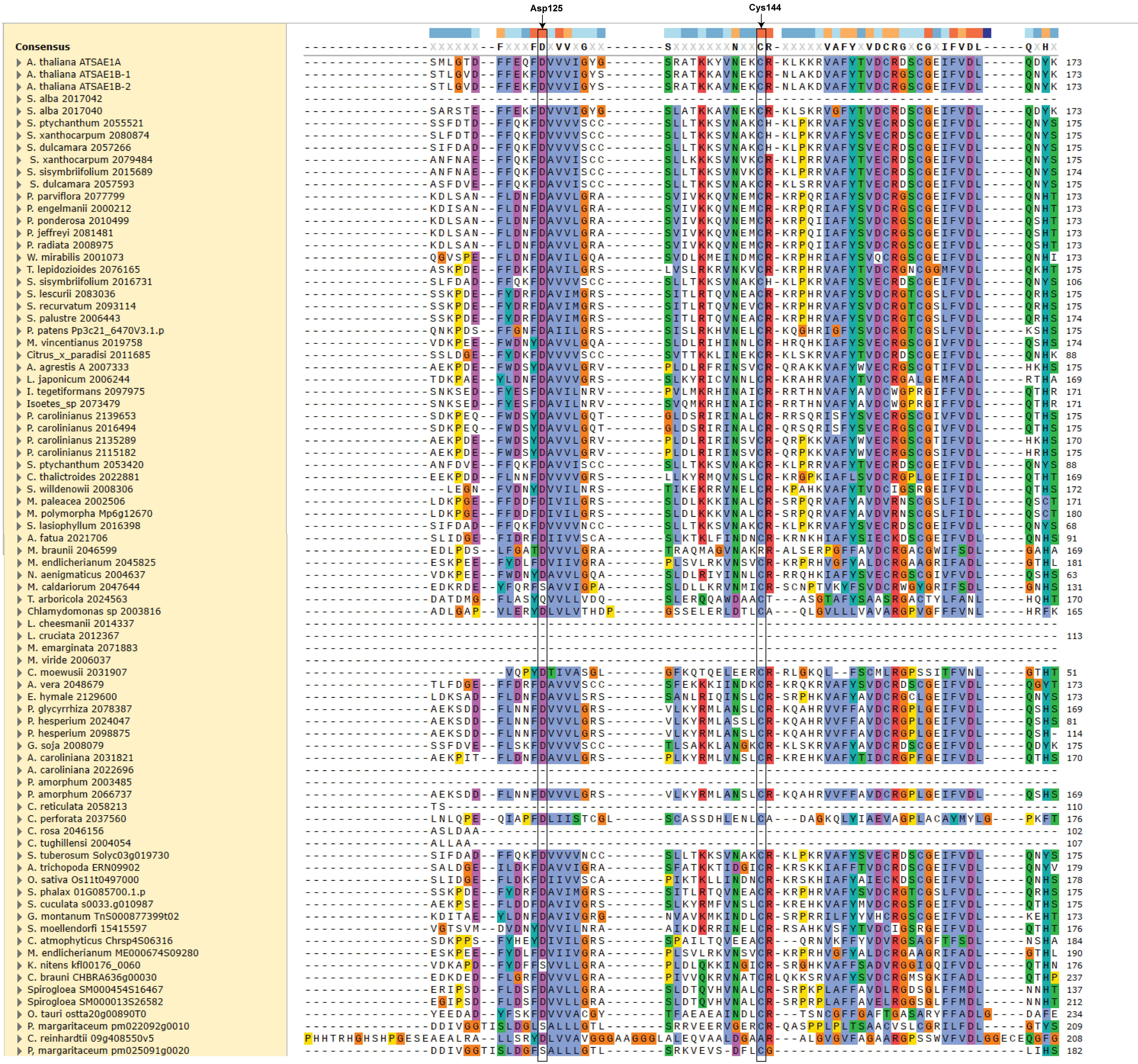

Figure S3 : Multiple Sequence Alignment of SAE1. The highlighted residues are the catalytic sites comprising of Adenylation site (Asp125) and catalytic domain (Cys144).

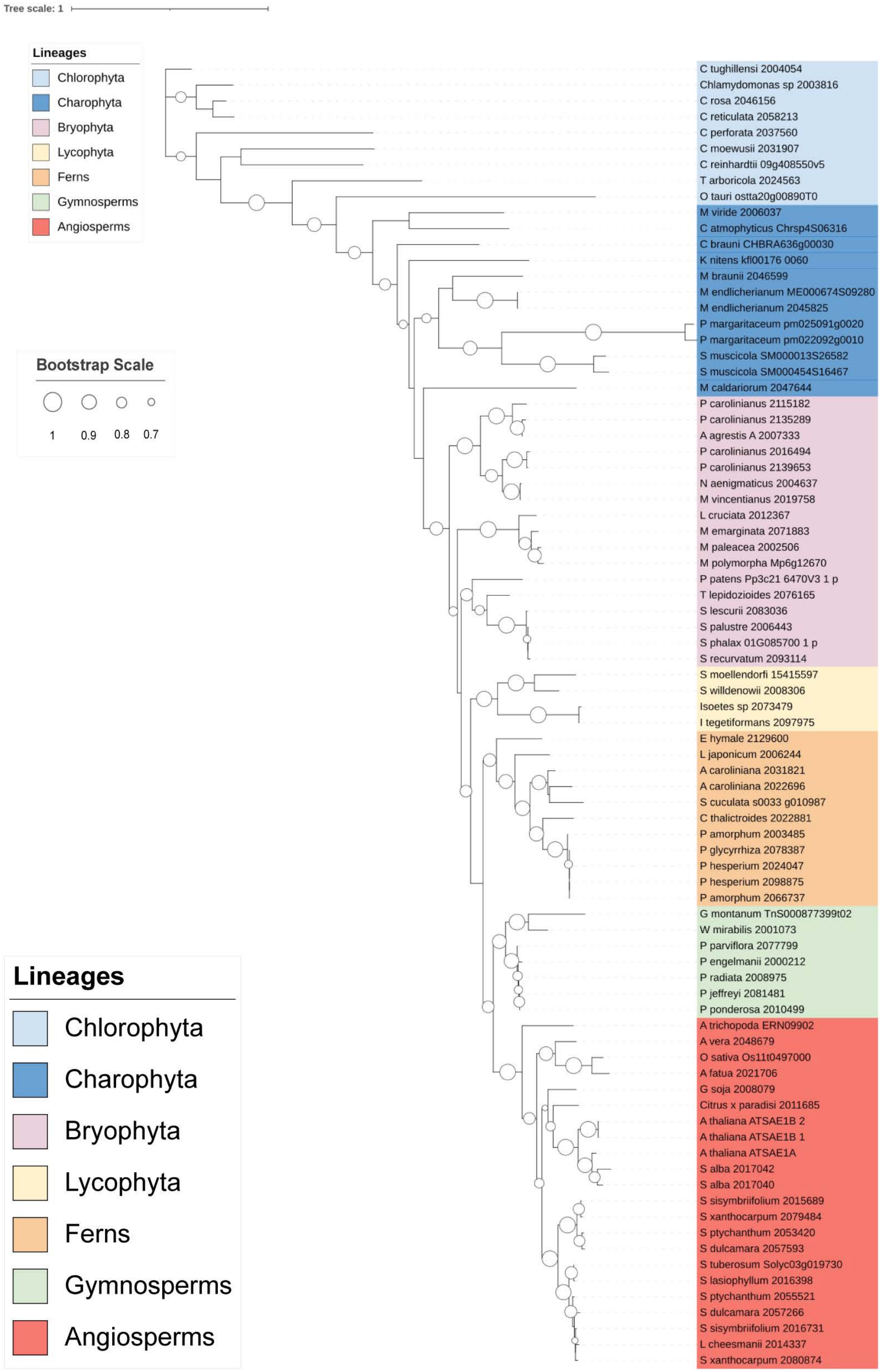

Figure S4: Phylogenetic analysis of SUMO Activating Enzyme 1 (SAE1). The corresponding alignments were obtained and processed as indicated in the text and the tree was constructed using PhyML. Bootstrap support was calculated for 1000 trees, and it is indicated in the branches with circles in which the size is proportional to the normalised bootstrap value (between 0 and 1). Only values higher than 0.7 are shown. The tree is drawn to scale, with branch lengths measuring the number of substitutions per site.

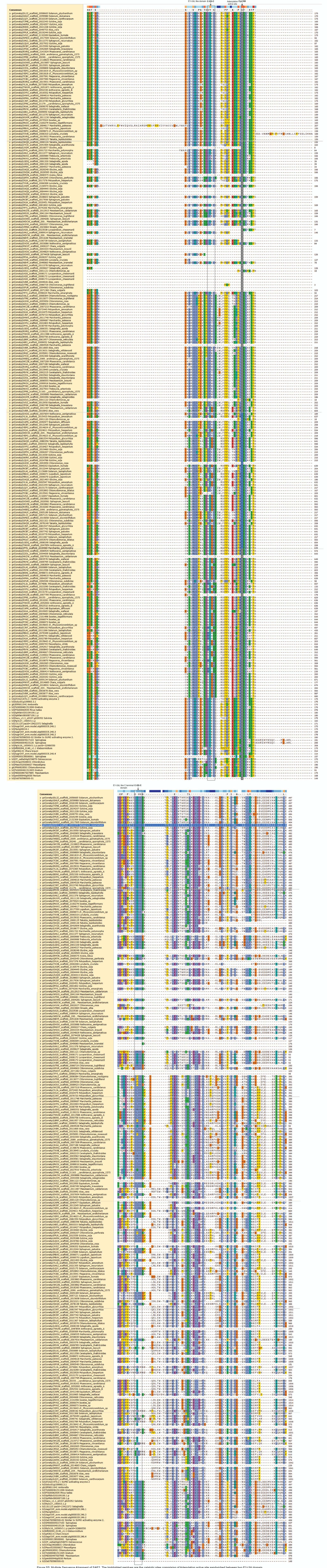

Figure S5: Multiple Sequence Alignment of SAE2. The highlighted residues are the catalytic sites comprising of Adenylation active site sandwiched between two E1-Ubl domain.

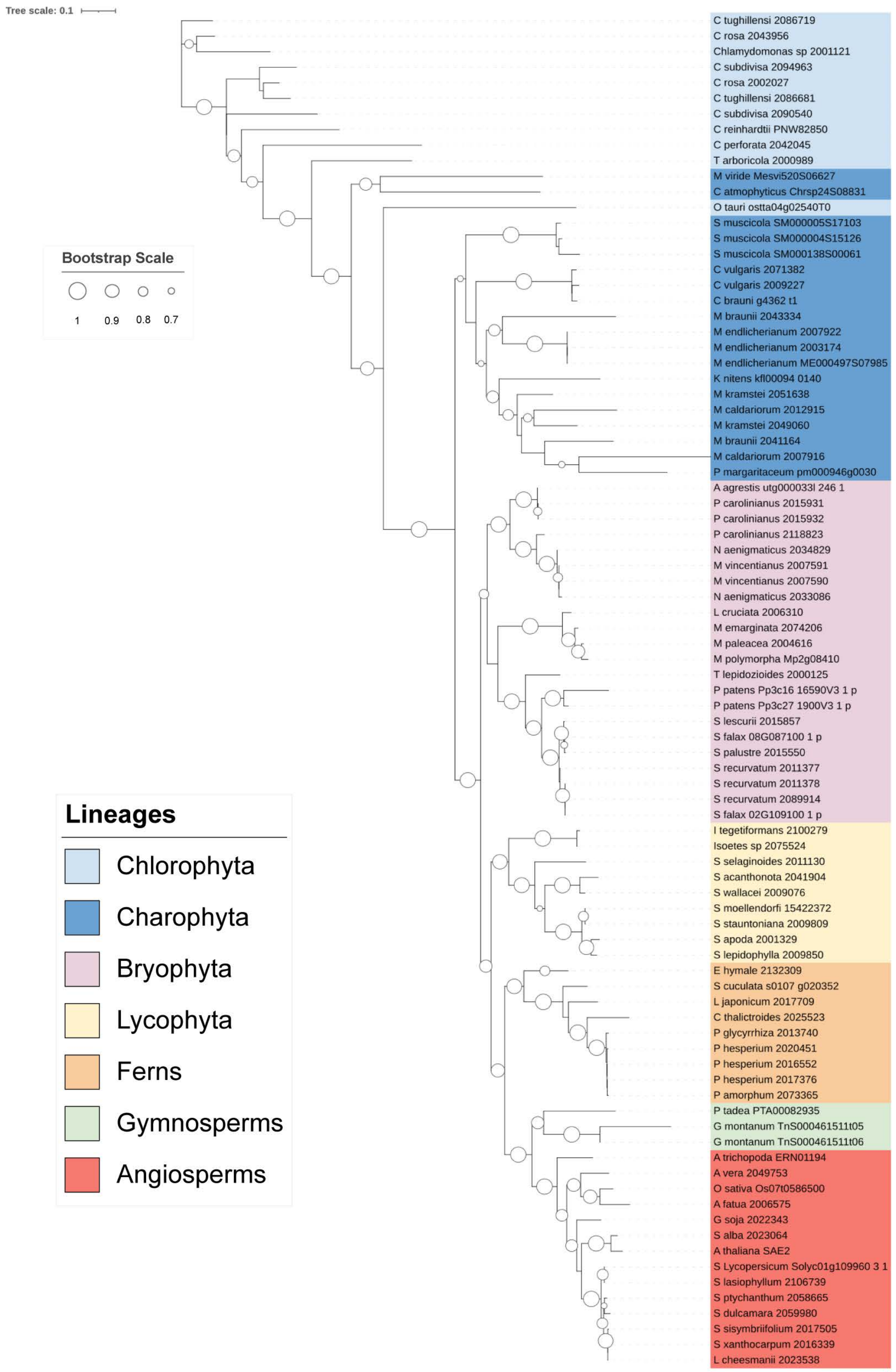

Figure S6: Phylogenetic analysis of SUMO Activating Enzyme 2 (SAE2). The corresponding alignments were obtained and processed as indicated in the text and the tree was constructed using PhyML. Bootstrap support was calculated for 1000 trees, and it is indicated in the branches with circles in which the size is proportional to the normalised bootstrap value (between 0 and 1). Only values higher than 0.7 are shown. The tree is drawn to scale, with branch lengths measuring the number of substitutions per site.

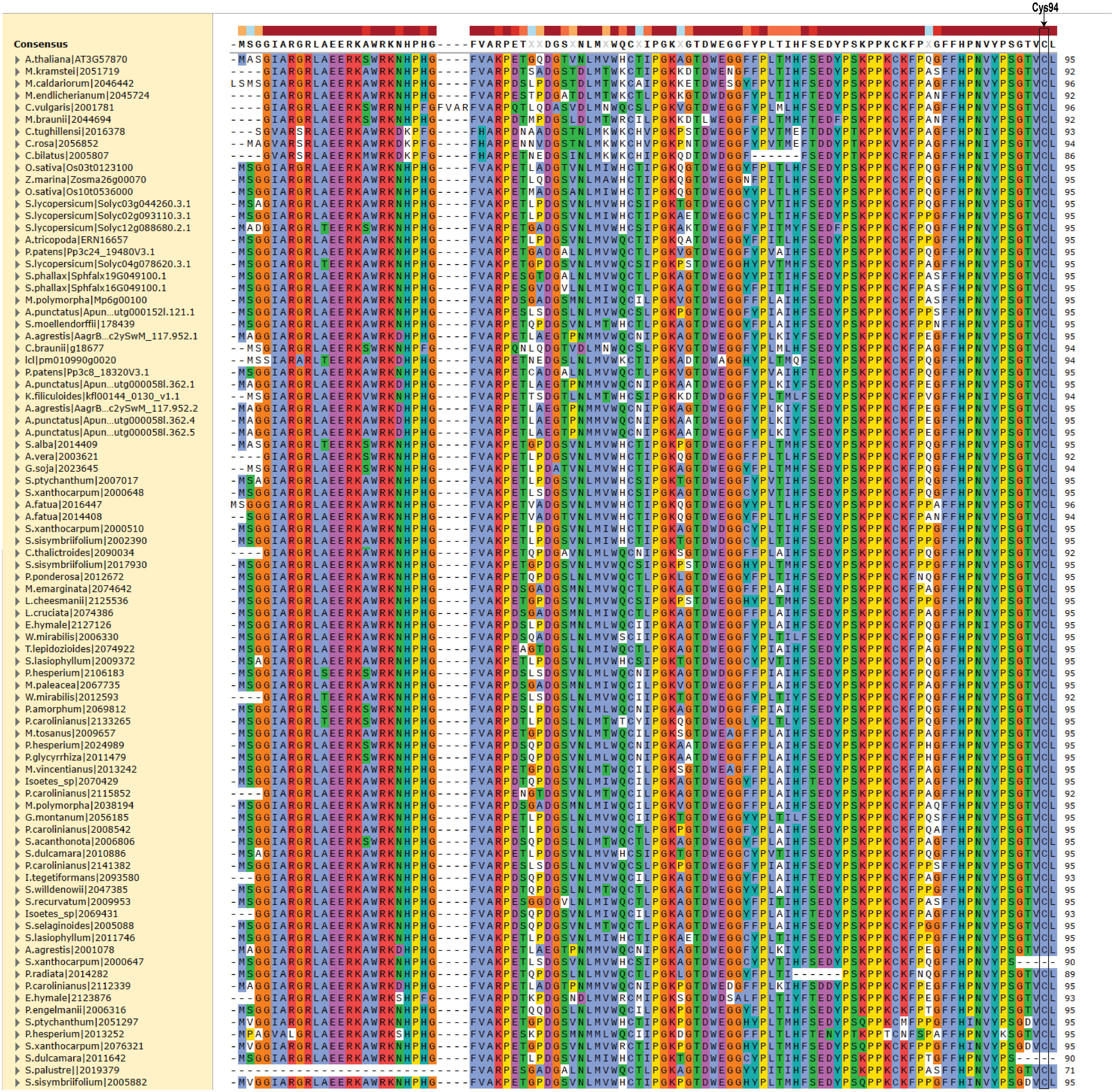

Figure S7: Multiple Sequence Alignment of SCE. The highlighted residue is the catalytic active site cysteine

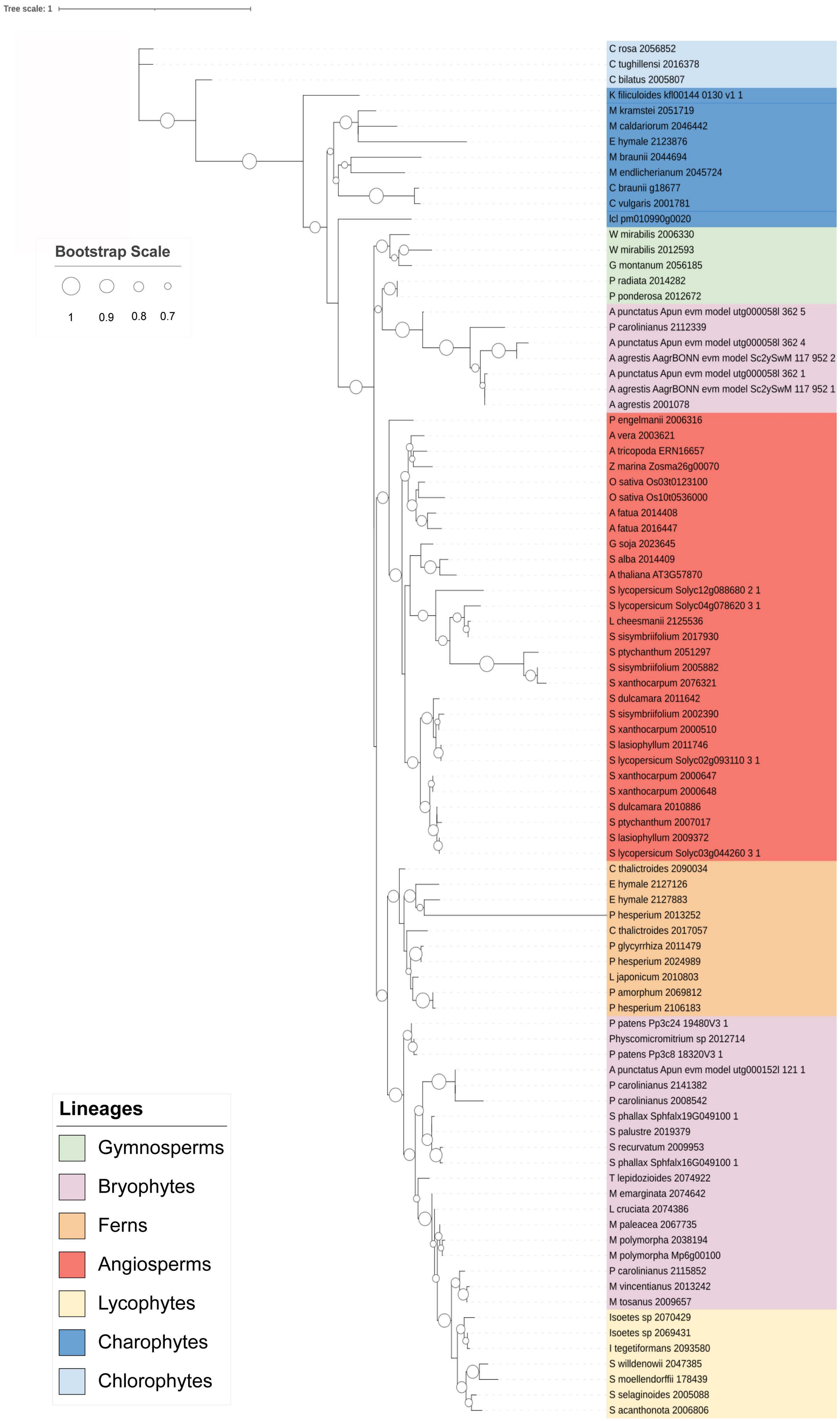

Figure S8: Phylogenetic analysis of SUMO Conjugating Enzyme (SCE1). The corresponding alignments were obtained and processed as indicated in the text and the tree was constructed using PhyML. Bootstrap support was calculated for 1000 trees, and it is indicated in the branches with circles in which the size is proportional to the normalised bootstrap value (between 0 and 1). Only values higher than 0.7 are shown. The tree is drawn to scale, with branch lengths measuring the number of substitutions per site.

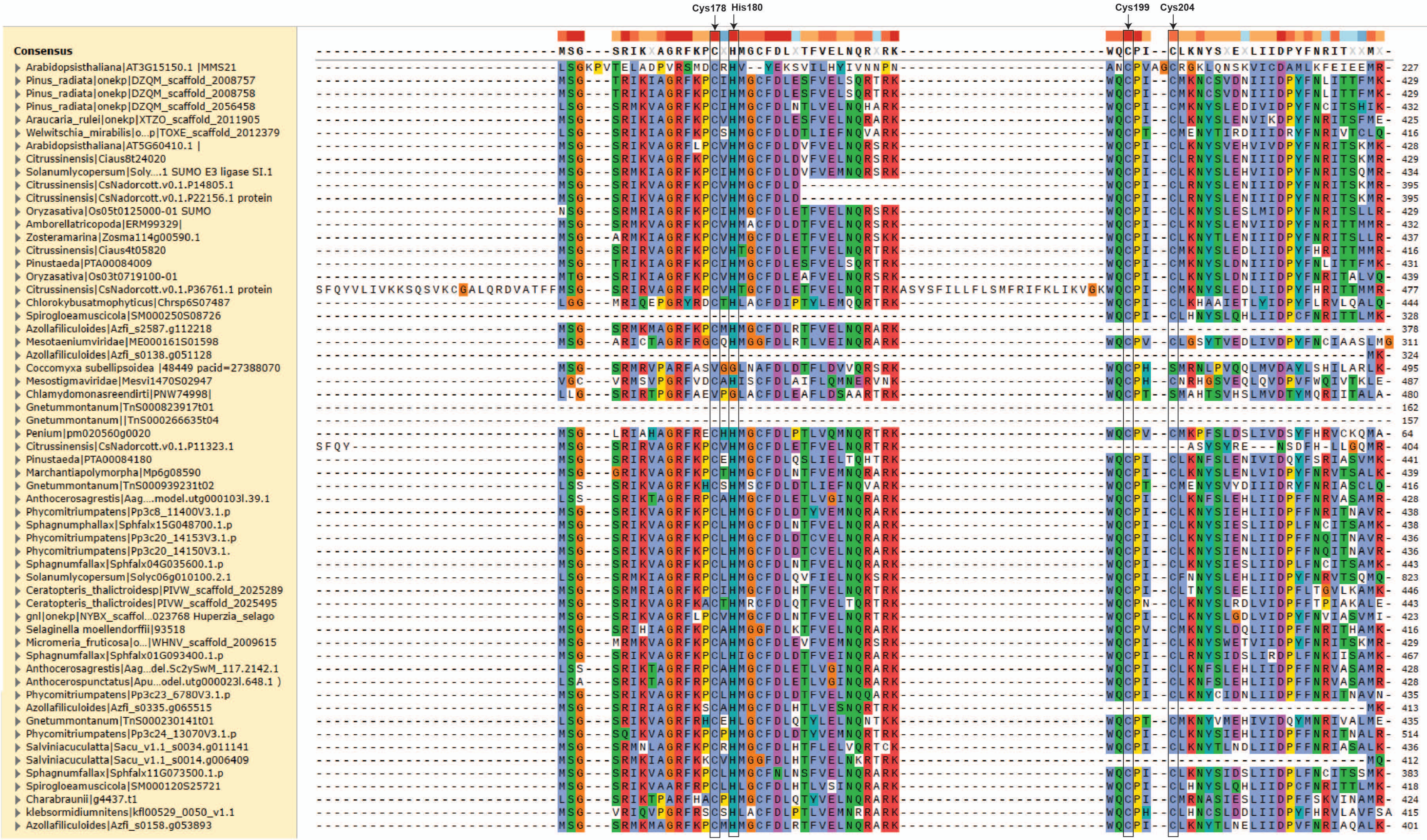

Figure S9 : Multiple Sequence Alignment of SIZ1. The highlighted residues are the conserved catalytic sites in the SP RING domain.

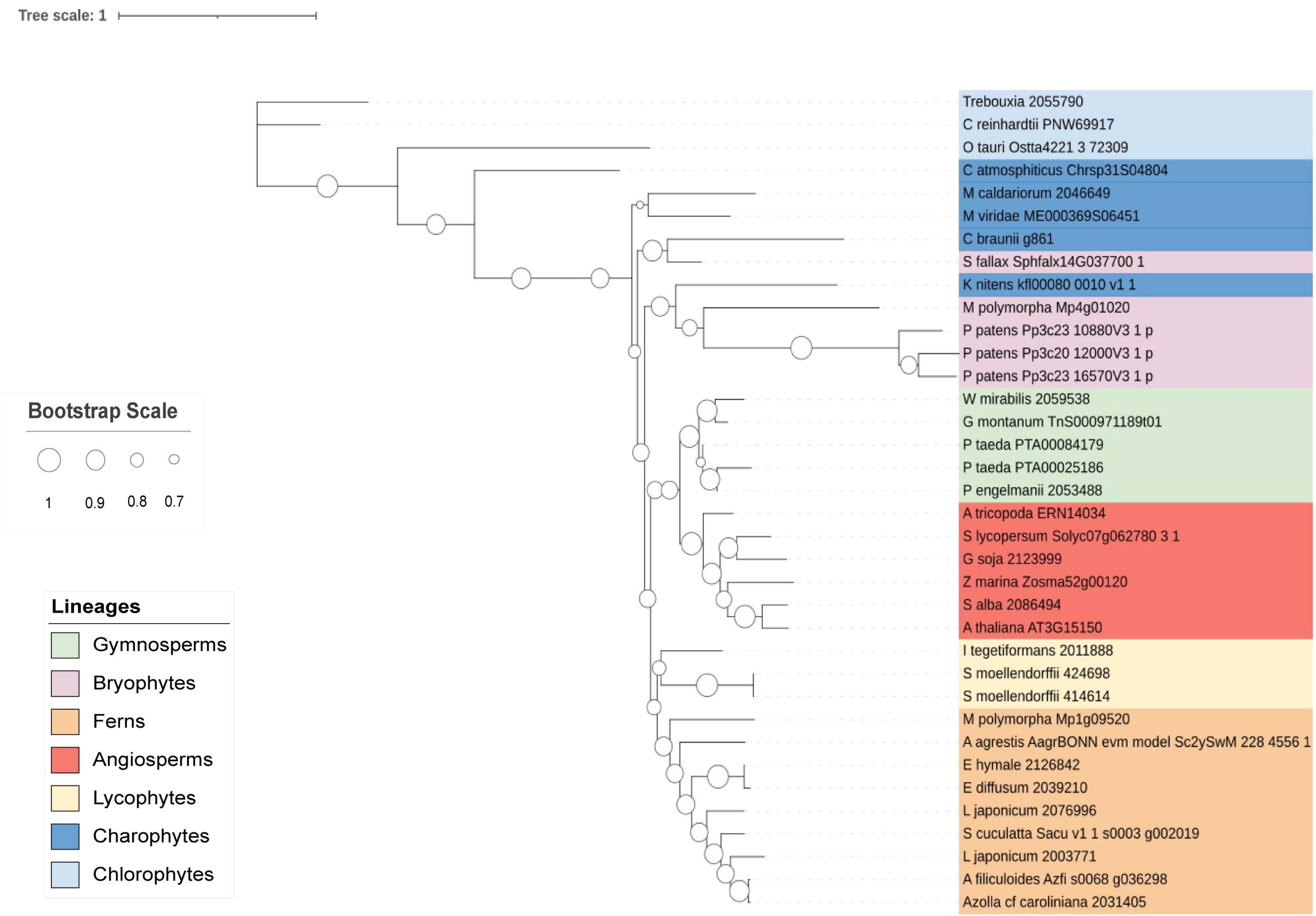

Figure S10: Phylogenetic analysis of SUMO E3 Ligase HPY2 (High Ploidy2). The corresponding alignments were obtained and processed as indicated in the text and the tree was constructed using PhyML. Bootstrap support was calculated for 1000 trees, and it is indicated in the branches with circles in which the size is proportional to the normalised bootstrap value (between 0 and 1). Only values higher than 0.7 are shown. The tree is drawn to scale, with branch lengths measuring the number of substitutions per site.

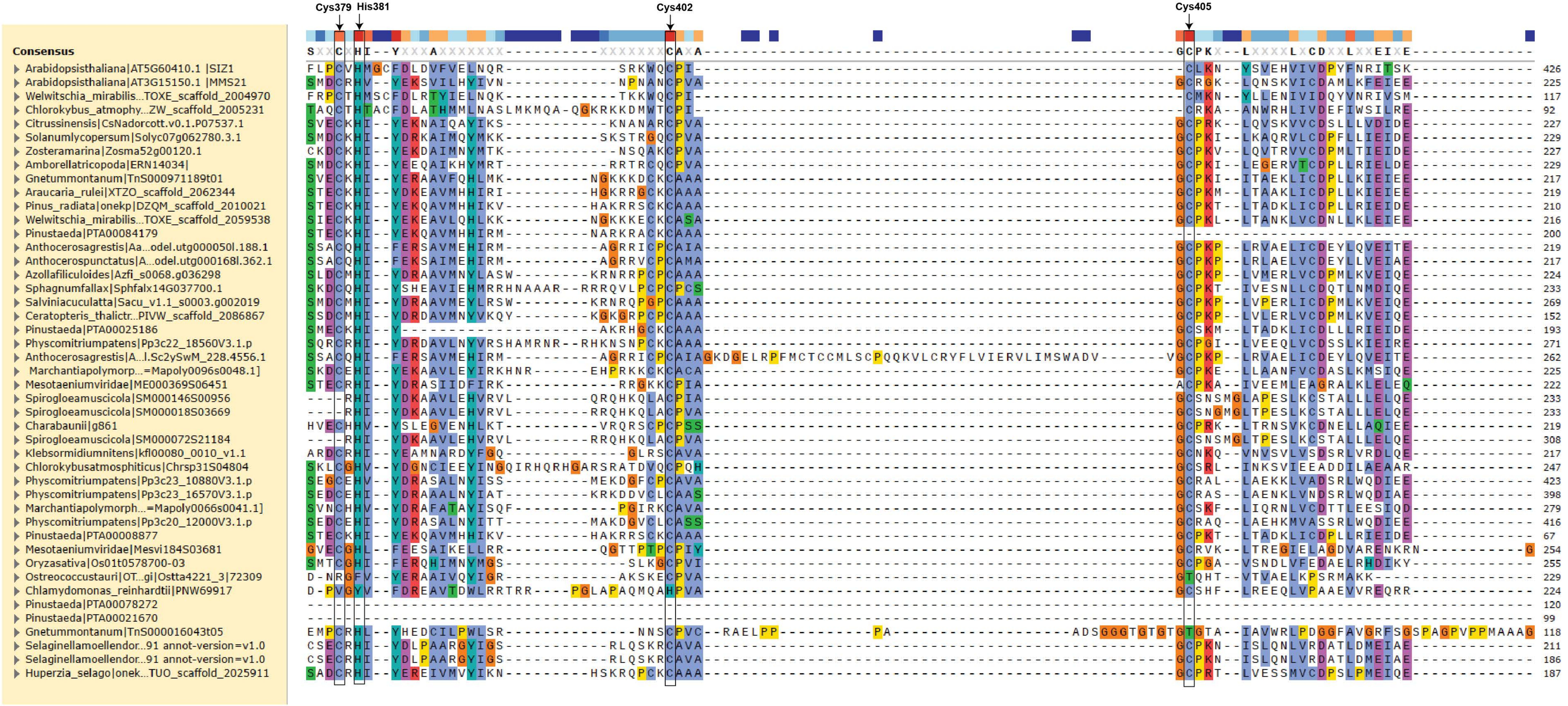

Figure S11: Multiple Sequence Alignment of HPY2. The highlighted residues are the conserved catalytic sites in the SP RING domain.

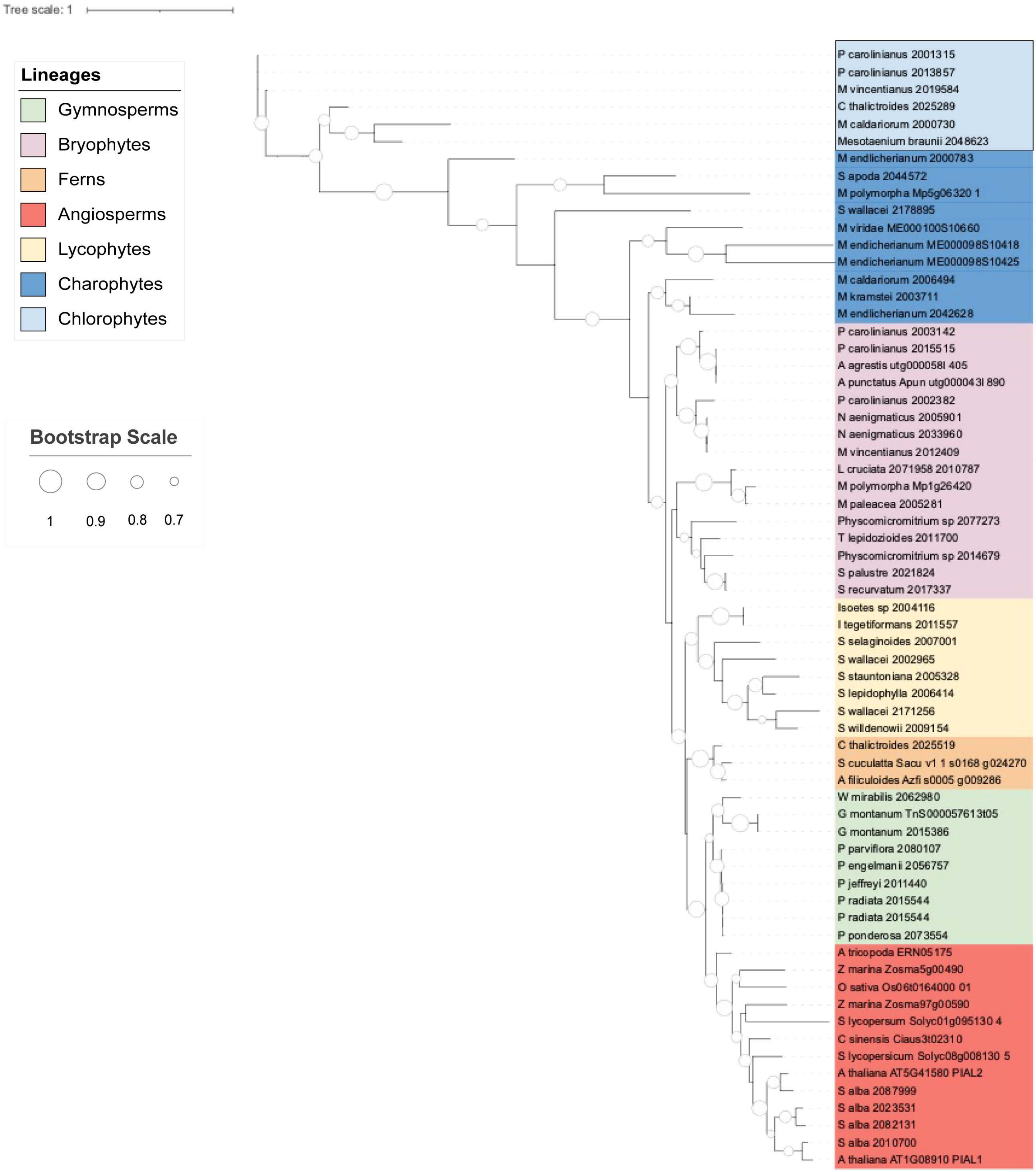

Figure S12: Phylogenetic analysis of SUMO E4 Ligase PIAL (PROTEIN INHIBITOR OF ACTIVATED STAT LIKE1). The corresponding alignments were obtained and processed as indicated in the text and the tree was constructed using PhyML. Bootstrap support was calculated for 1000 trees, and it is indicated in the branches with circles in which the size is proportional to the normalised bootstrap value (between 0 and 1). Only values higher than 0.7 are shown. The tree is drawn to scale, with branch lengths measuring the number of substitutions per site.

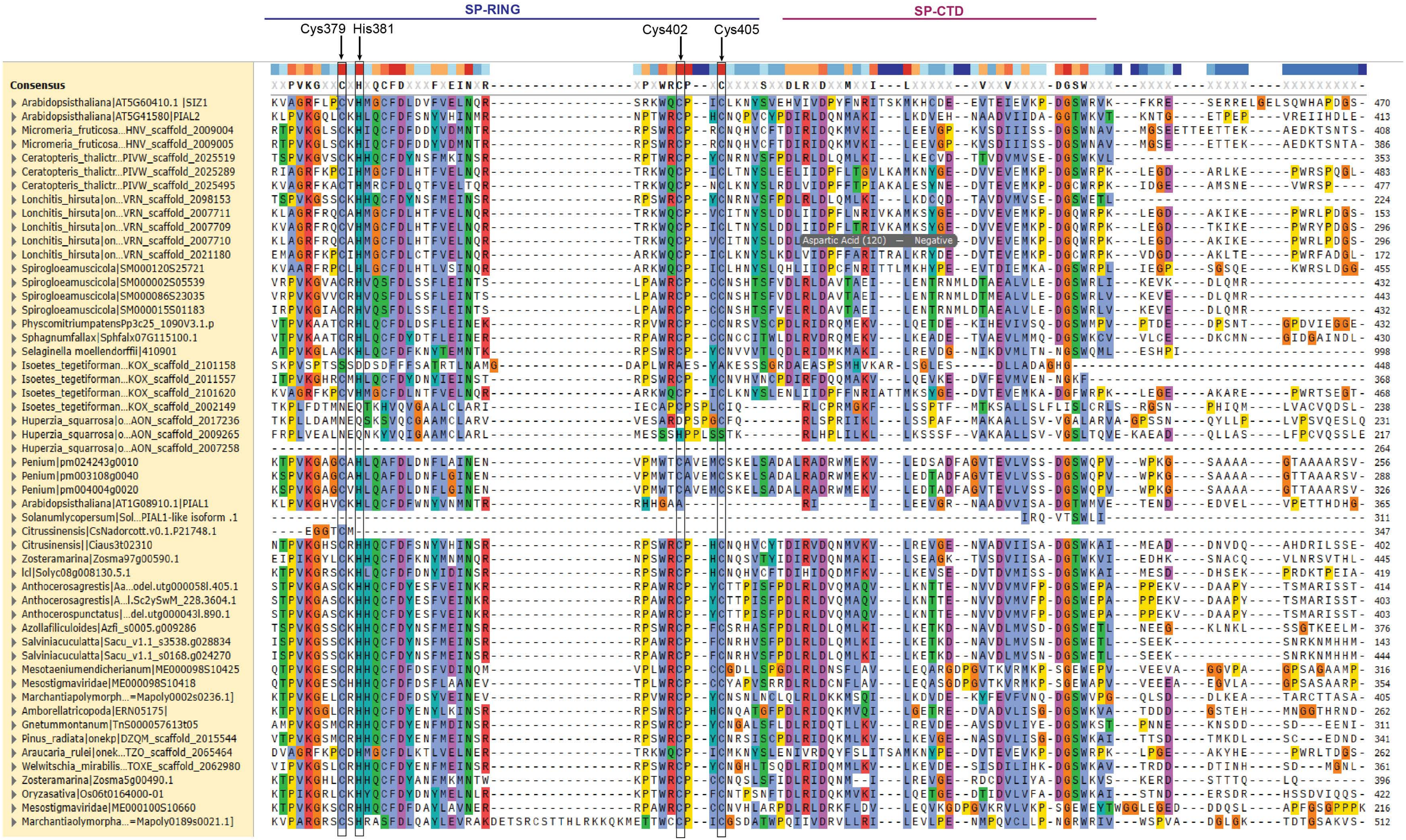

Fig S13: Multiple sequence alignment of PIAL highlighting the conserved SP-RING domain and SP-CTD domain. The conserved residues at the catalytic SP domain (Cys 379, His381, Cys402 and Cys405) are marked with arrows.

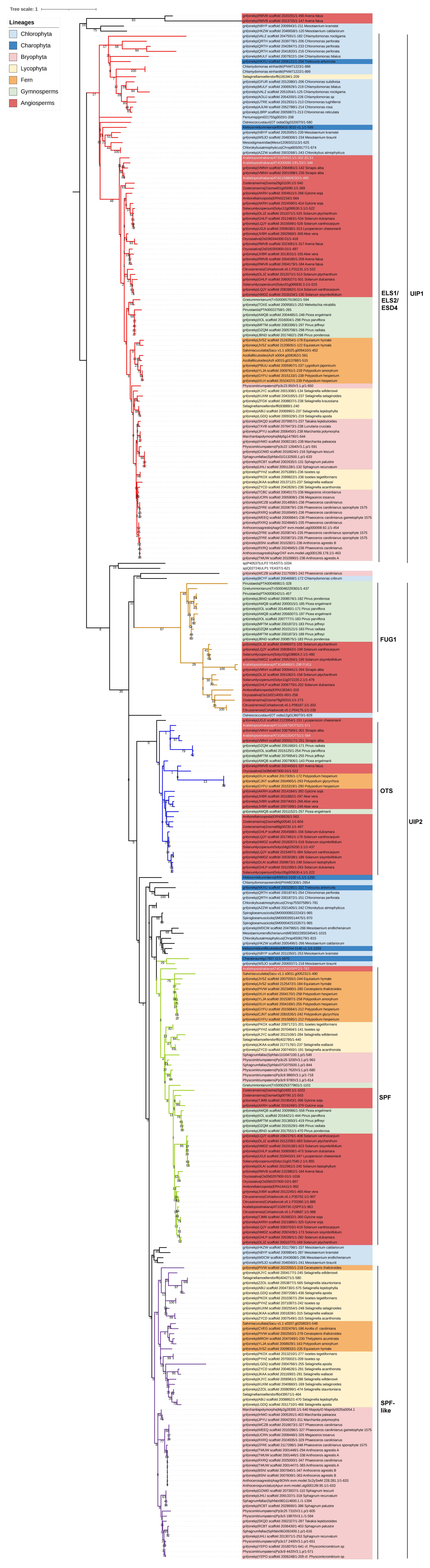

Figure S14: Phylogenetic analysis of UIP type proteases showing the evolution of UIP1 and UIP2 type proteases across different plant lineages. The corresponding alignments were obtained and processed as indicated in the text and the tree was constructed using PhyML. Bootstrap support was calculated for 1000 trees, and it is indicated in the branches, with values >0.5 shownb in ticker lines. The tree is drawn to scale, with branch lengths measuring the number of substitutions per site. The tree is rooted to the midpoint.

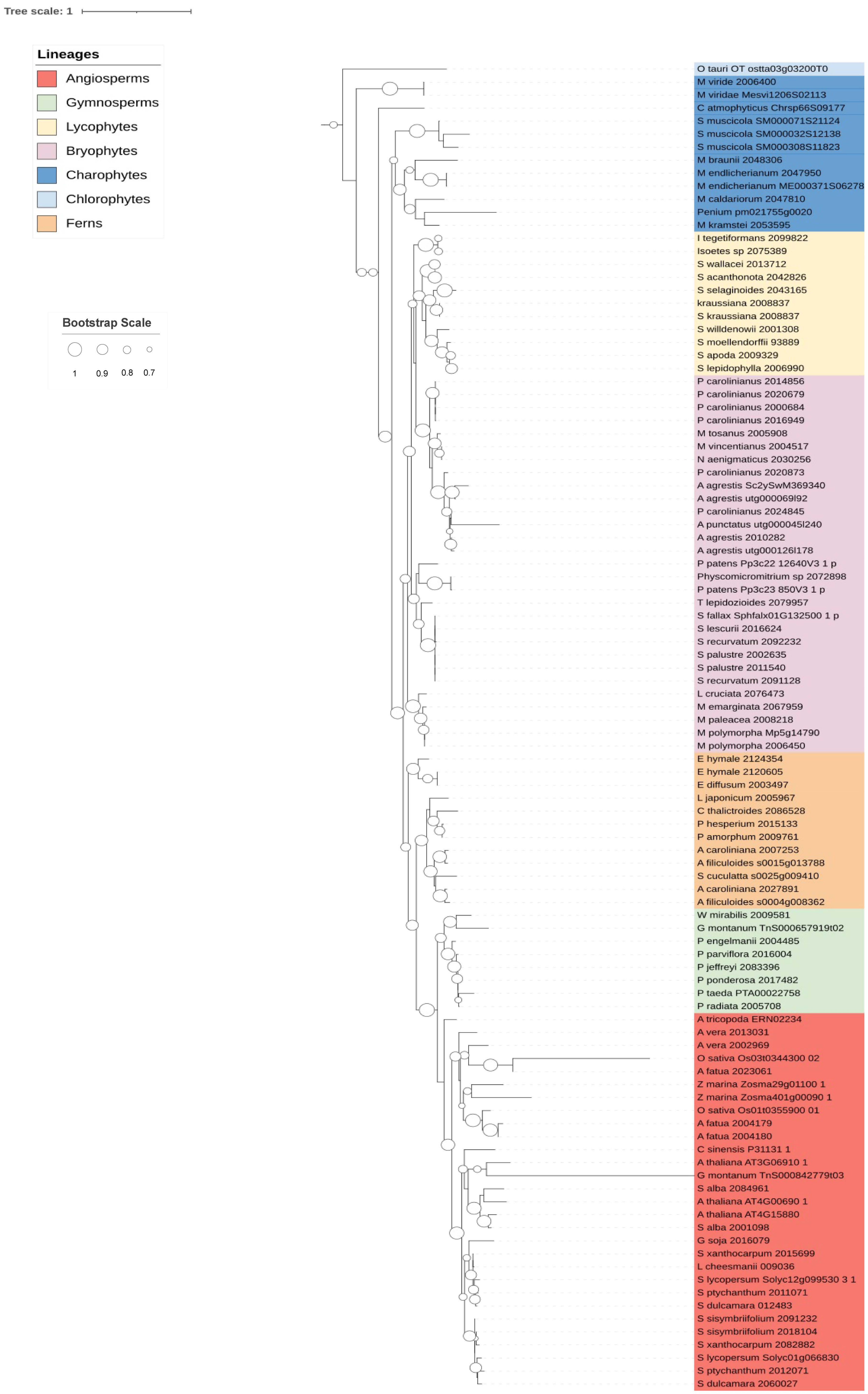

Figure S15: Phylogenetic analysis of SUMO protease UIP1 (Ubiquitin-like protease1). The corresponding alignments were obtained and processed as indicated in the text and the tree was constructed using PhyML. Bootstrap support was calculated for 1000 trees, and it is indicated in the branches with circles in which the size is proportional to the normalised bootstrap value (between 0 and 1). Only values higher than 0.7 are shown. The tree is drawn to scale, with branch lengths measuring the number of substitutions per site.

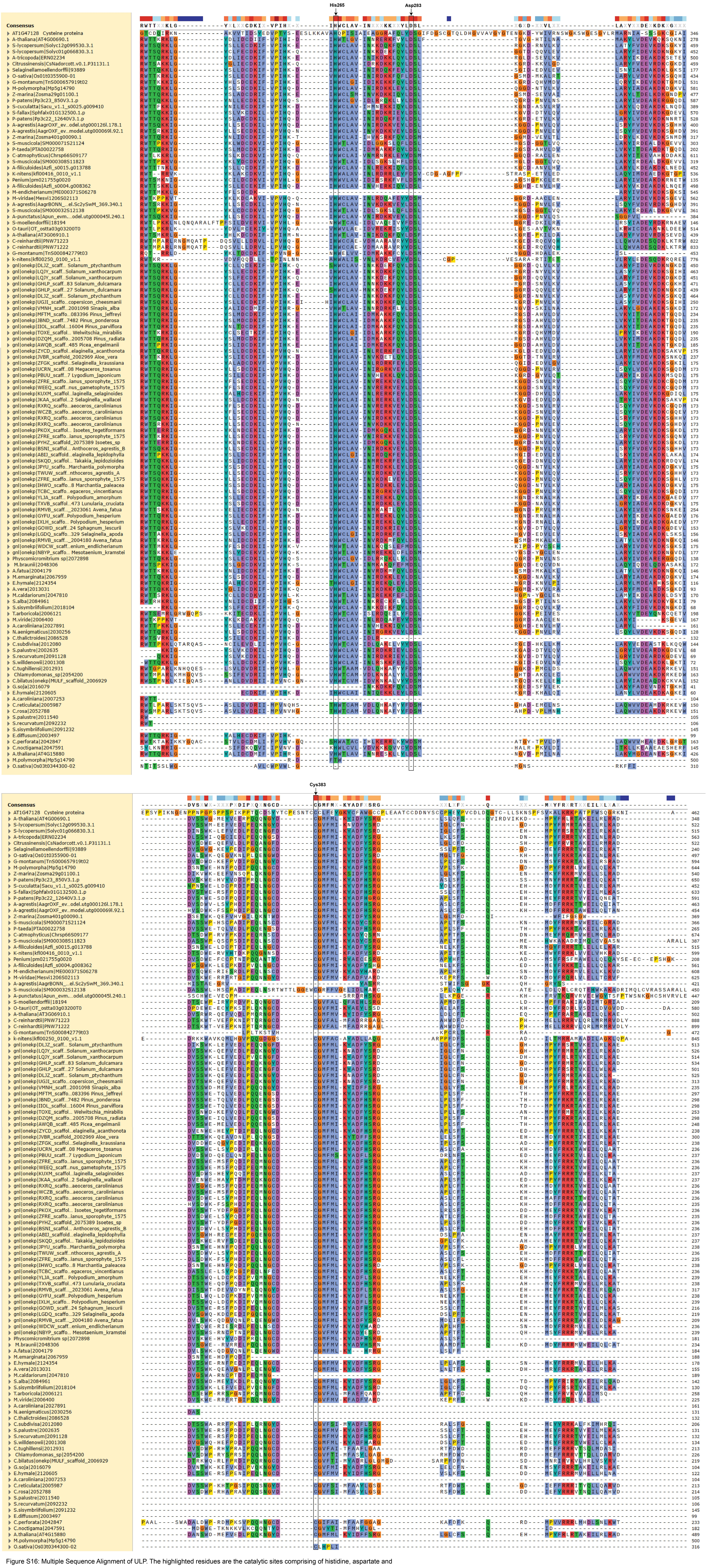

Figure S16: Multiple Sequence Alignment of ULP. The highlighted residues are the catalytic sites comprising of histidine, aspartate and

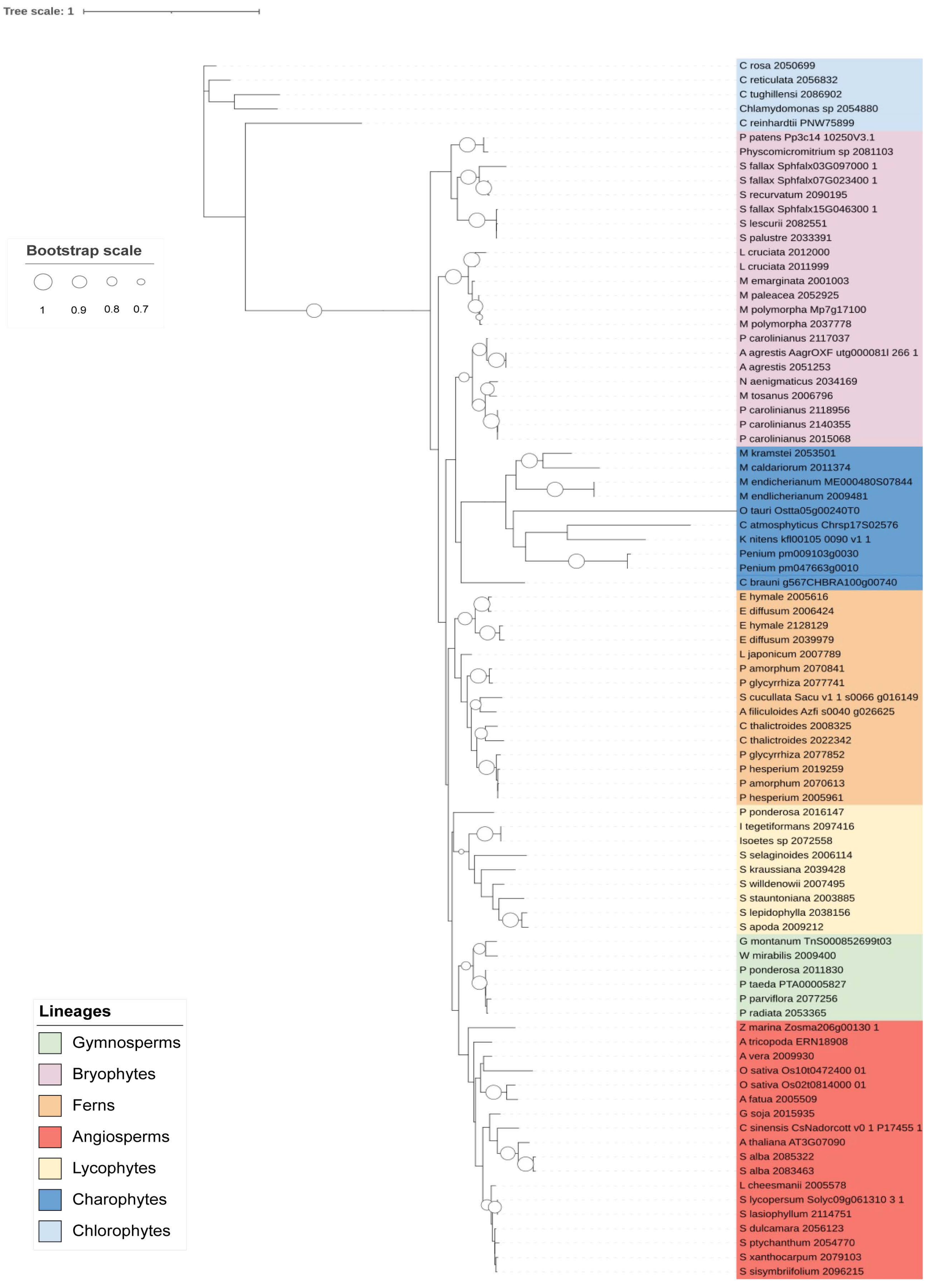

Figure S17: Phylogenetic analysis of SUMO protease DeSI1 (DeSumoylating Isopeptidase 1). The corresponding alignments were obtained and processed as indicated in the text and the tree was constructed using PhyML. Bootstrap support was calculated for 1000 trees, and it is indicated in the branches with circles in which the size is proportional to the normalised bootstrap value (between 0 and 1). Only values higher than 0.7 are shown. The tree is drawn to scale, with branch lengths measuring the number of substitutions per site.

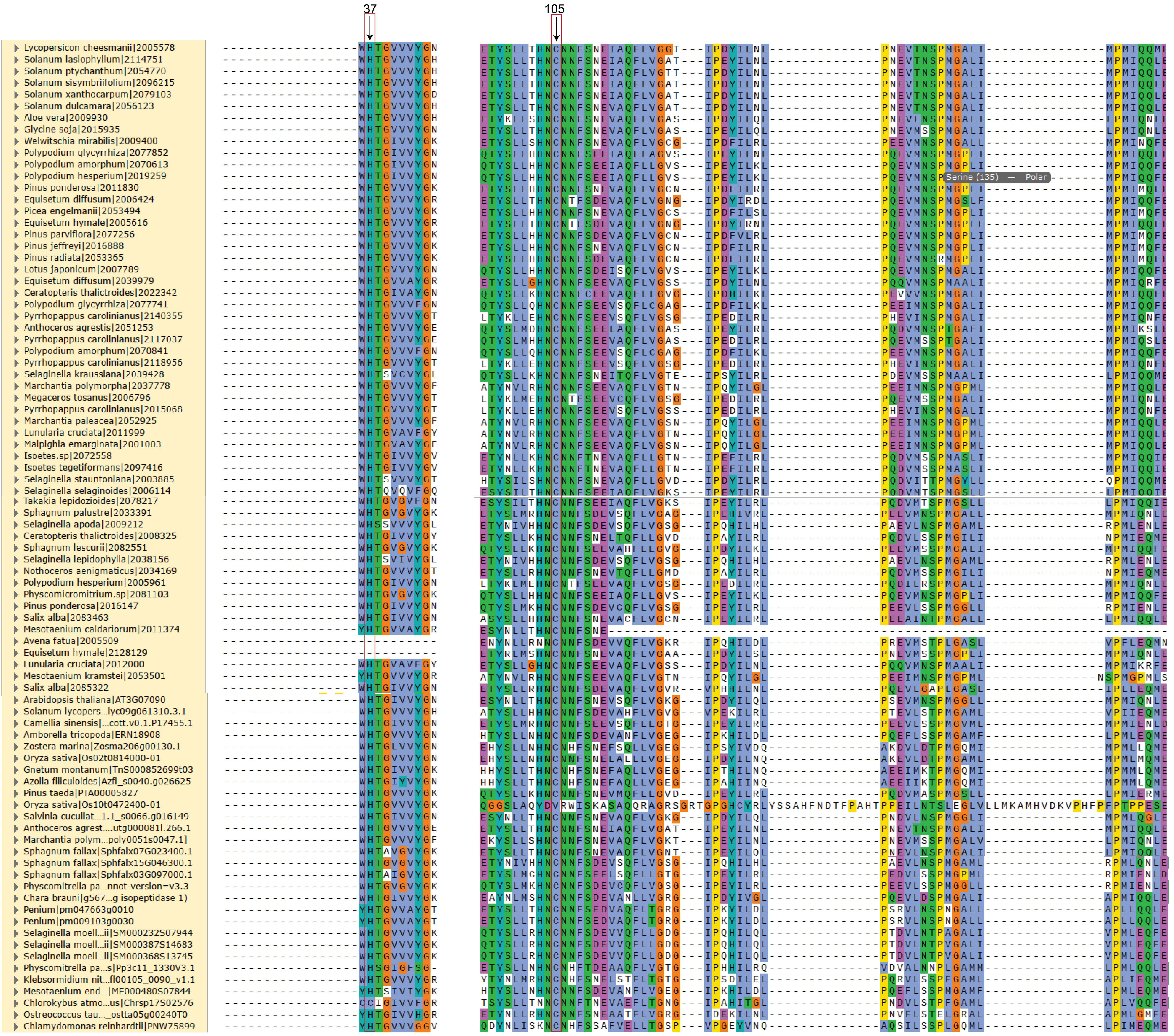

Figure S18: Multiple Sequence Alignment of DESI1. The highlighted residues are the catalytic sites omprising of cysteine and histidine

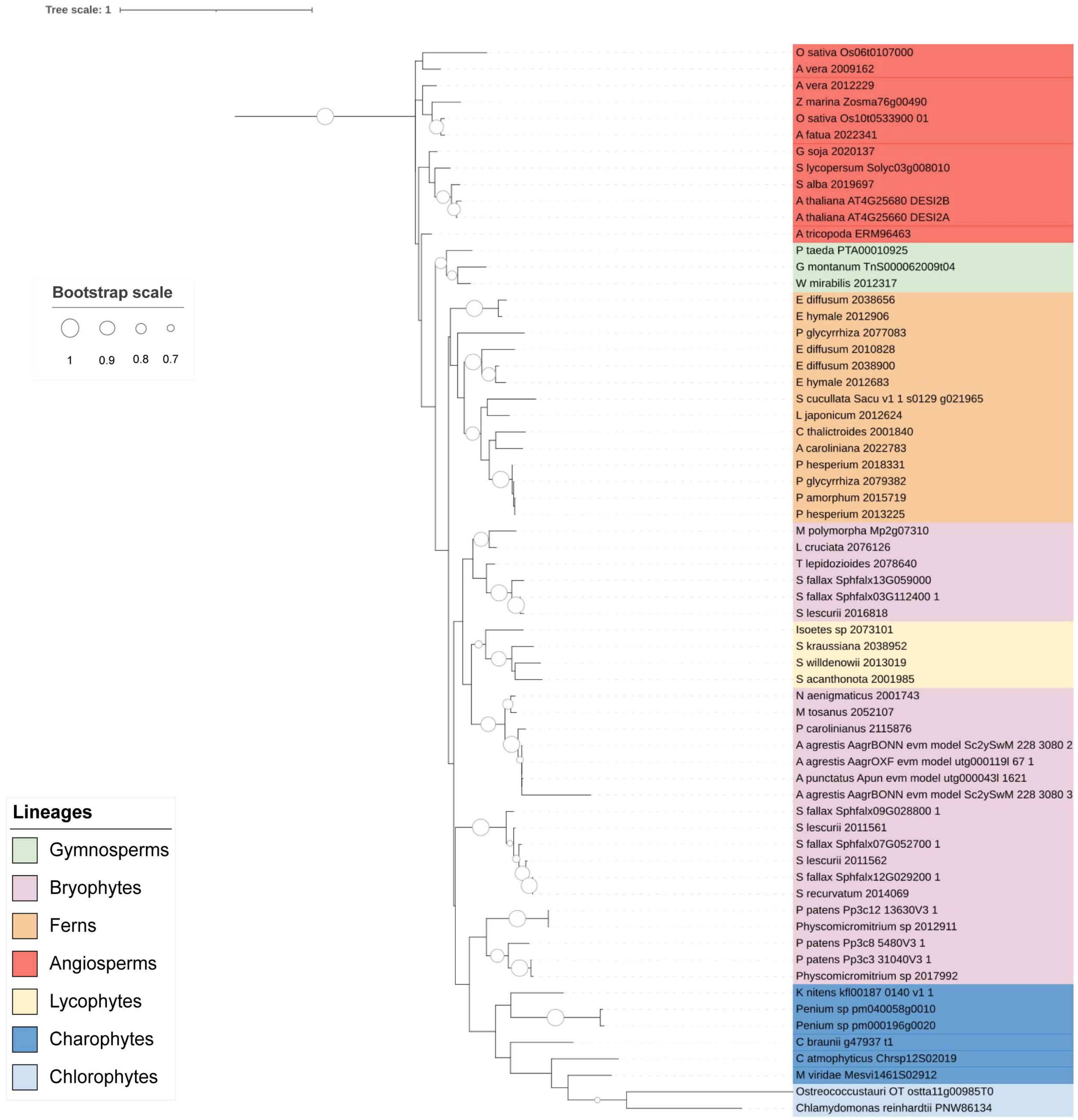

Figure S19: Phylogenetic analysis of SUMO protease DeSI2 (DeSumoylating Isopeptidase 2). The corresponding alignments were obtained and processed as indicated in the text and the tree was constructed using PhyML. Bootstrap support was calculated for 1000 trees, and it is indicated in the branches with circles in which the size is proportional to the normalised bootstrap value (between 0 and 1). Only values higher than 0.7 are shown. The tree is drawn to scale, with branch lengths measuring the number of substitutions per site.

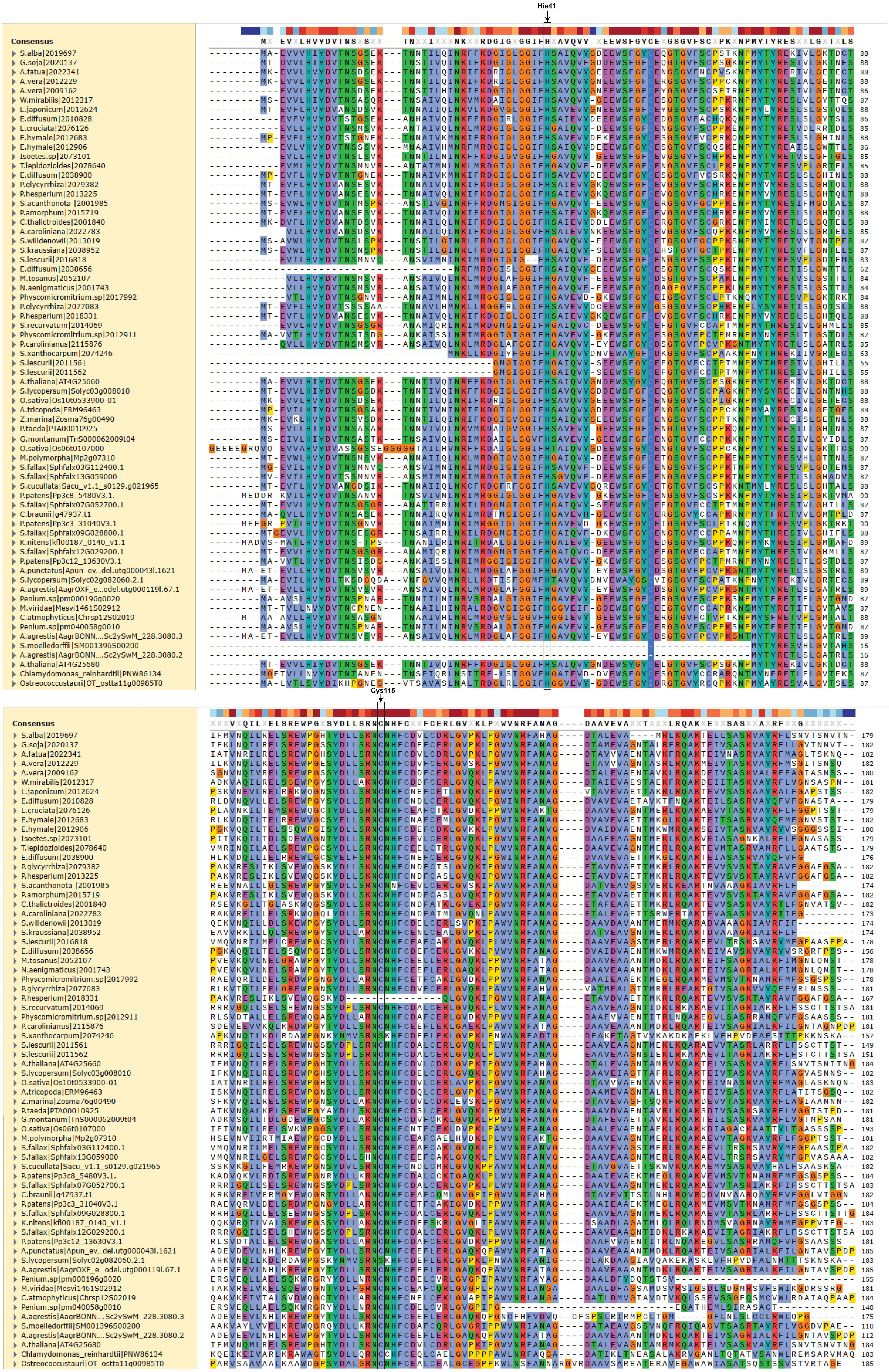

Figure S20: Multiple Sequence Alignment of DeSI2. The highlighted residues are the catalytic sites comprising of histidine and cysteine.

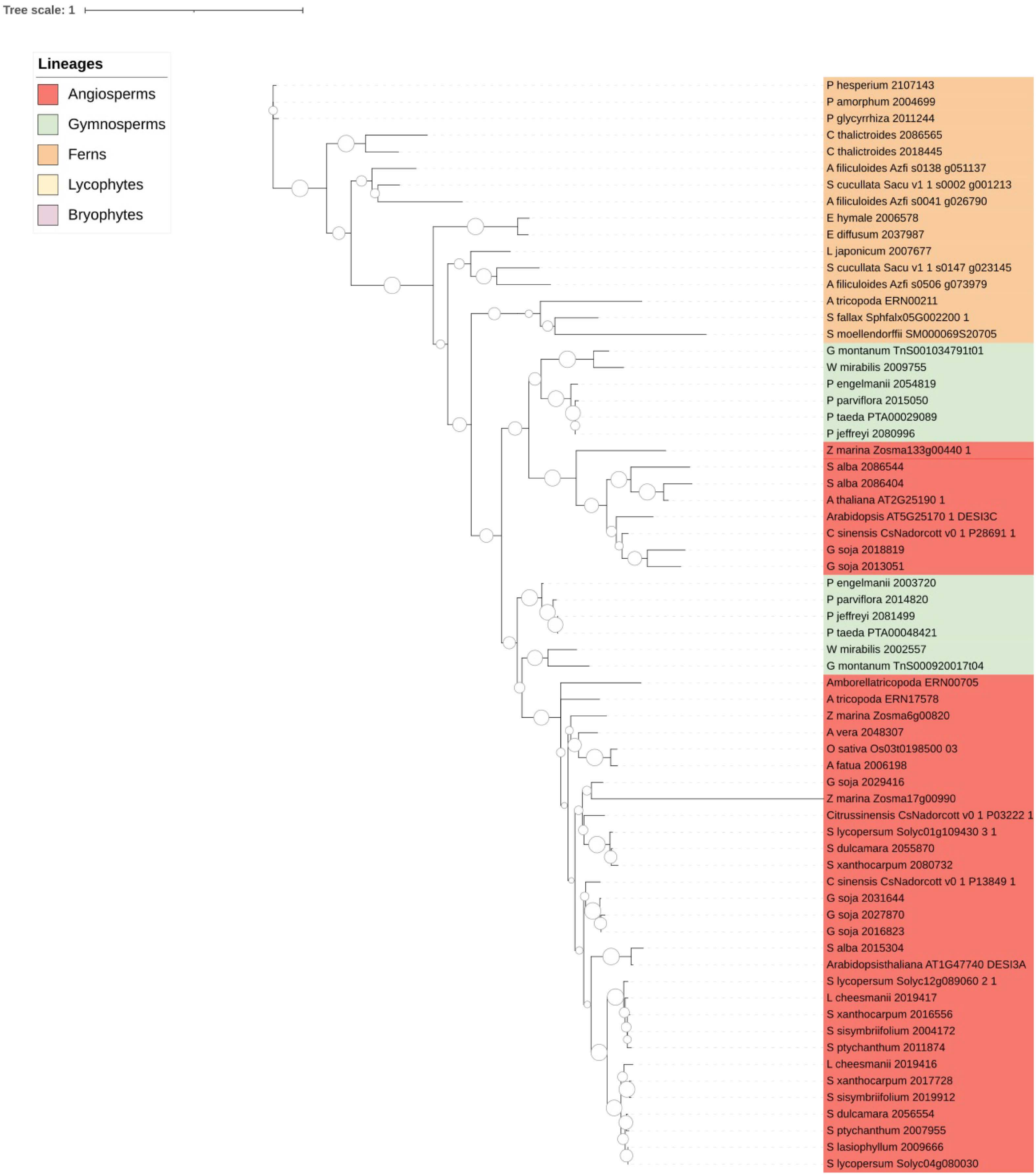

Figure S21: Phylogenetic analysis of SUMO protease DeSI3 (DeSumoylating Isopeptidase 3). The corresponding alignments were obtained and processed as indicated in the text and the tree was constructed using PhyML. Bootstrap support was calculated for 1000 trees, and it is indicated in the branches with circles in which the size is proportional to the normalised bootstrap value (between 0 and 1). Only values higher than 0.7 are shown. The tree is drawn to scale, with branch lengths measuring the number of substitutions per site.

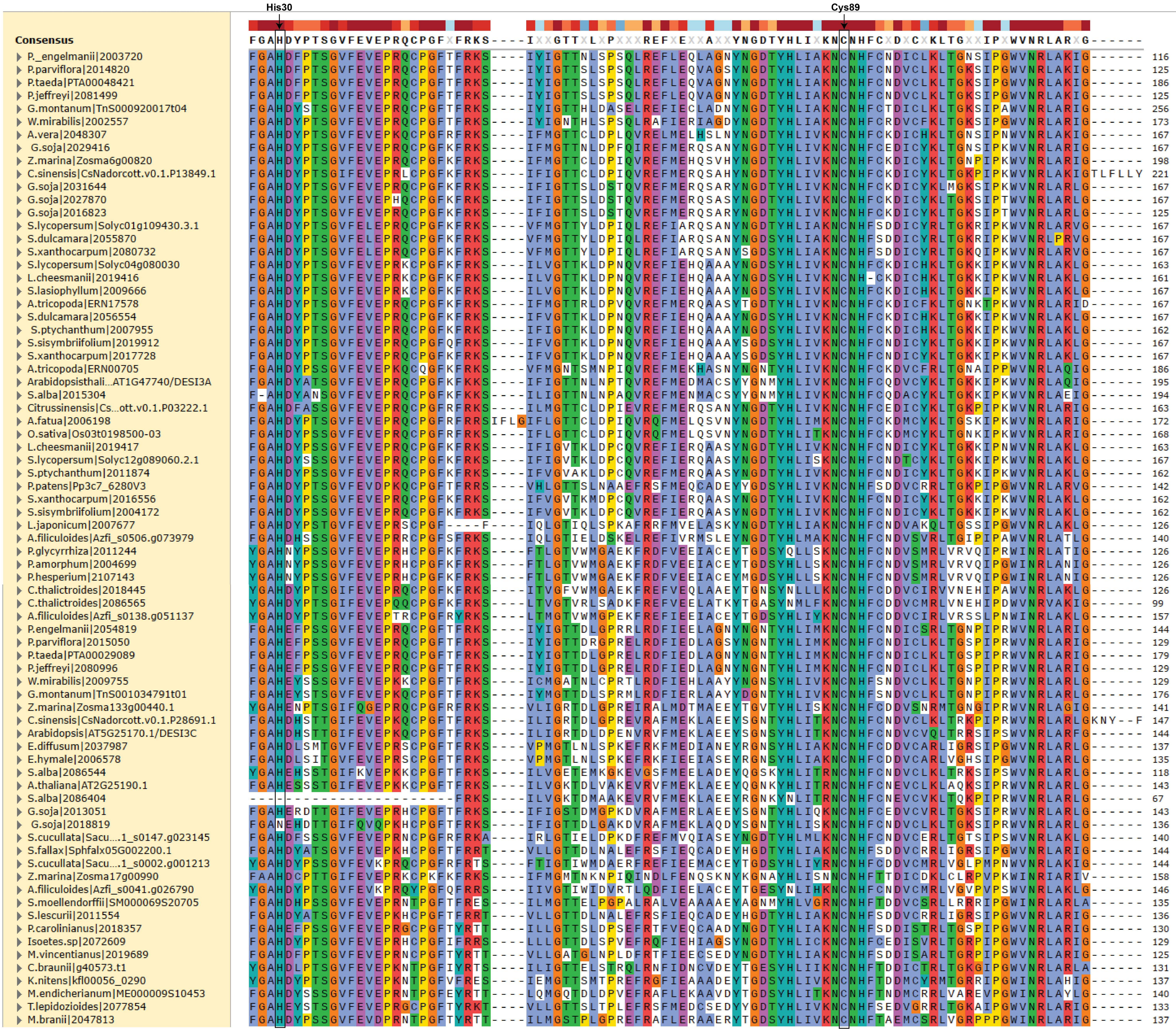

Figure S22: Multiple Sequence Alignment of DeSI3. The highlighted residues are the catalytic sites comprising of histidine and cysteine.

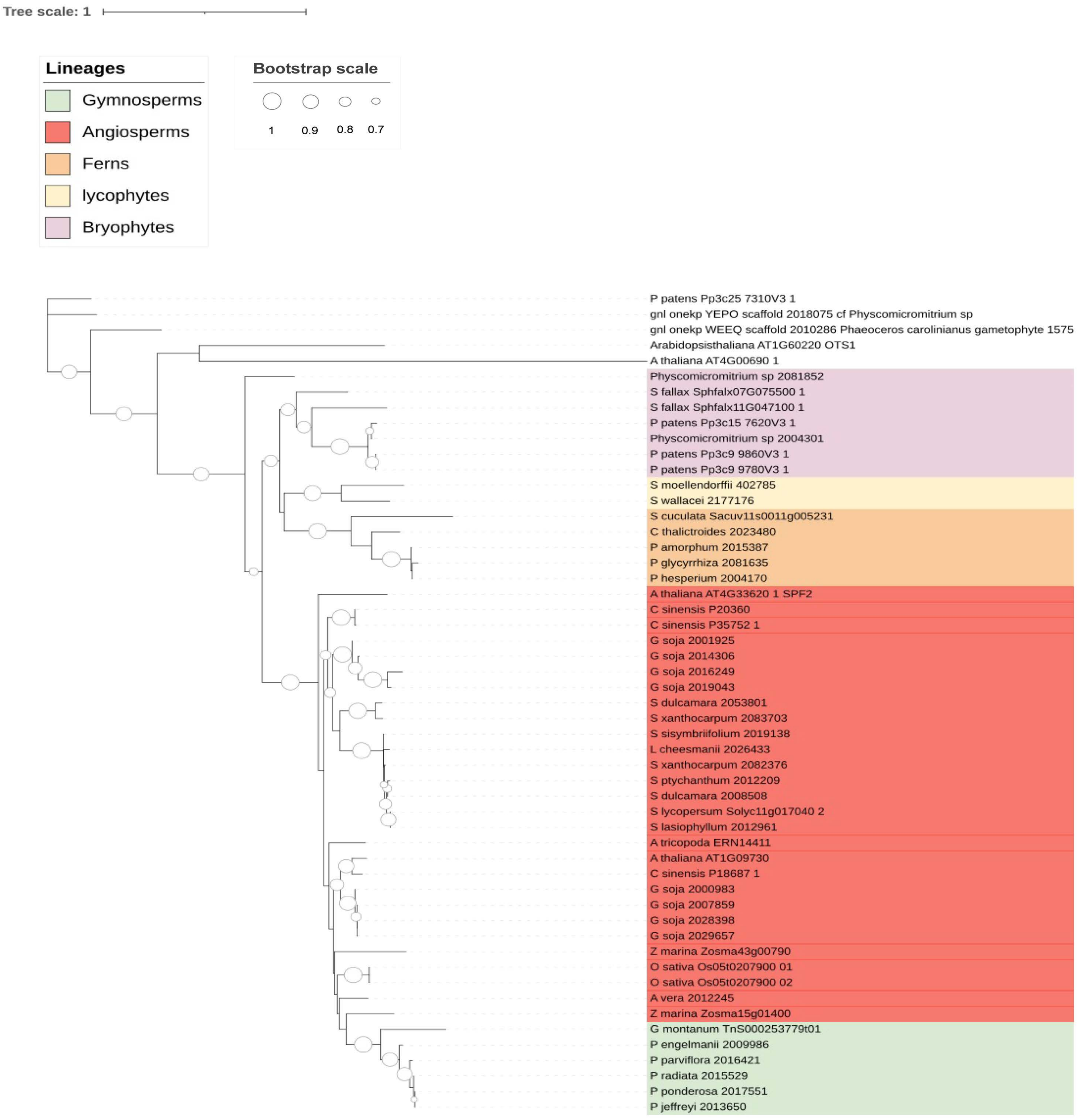

Figure S23: Phylogenetic analysis of SUMO protease SPF (SUMO Protease related to Fertility).. The corresponding alignments were obtained and processed as indicated in the text and the tree was constructed using PhyML. Bootstrap support was calculated for 1000 trees, and it is indicated in the branches with circles in which the size is proportional to the normalised bootstrap value (between 0 and 1). Only values higher than 0.7 are shown. The tree is drawn to scale, with branch lengths measuring the number of substitutions per site.

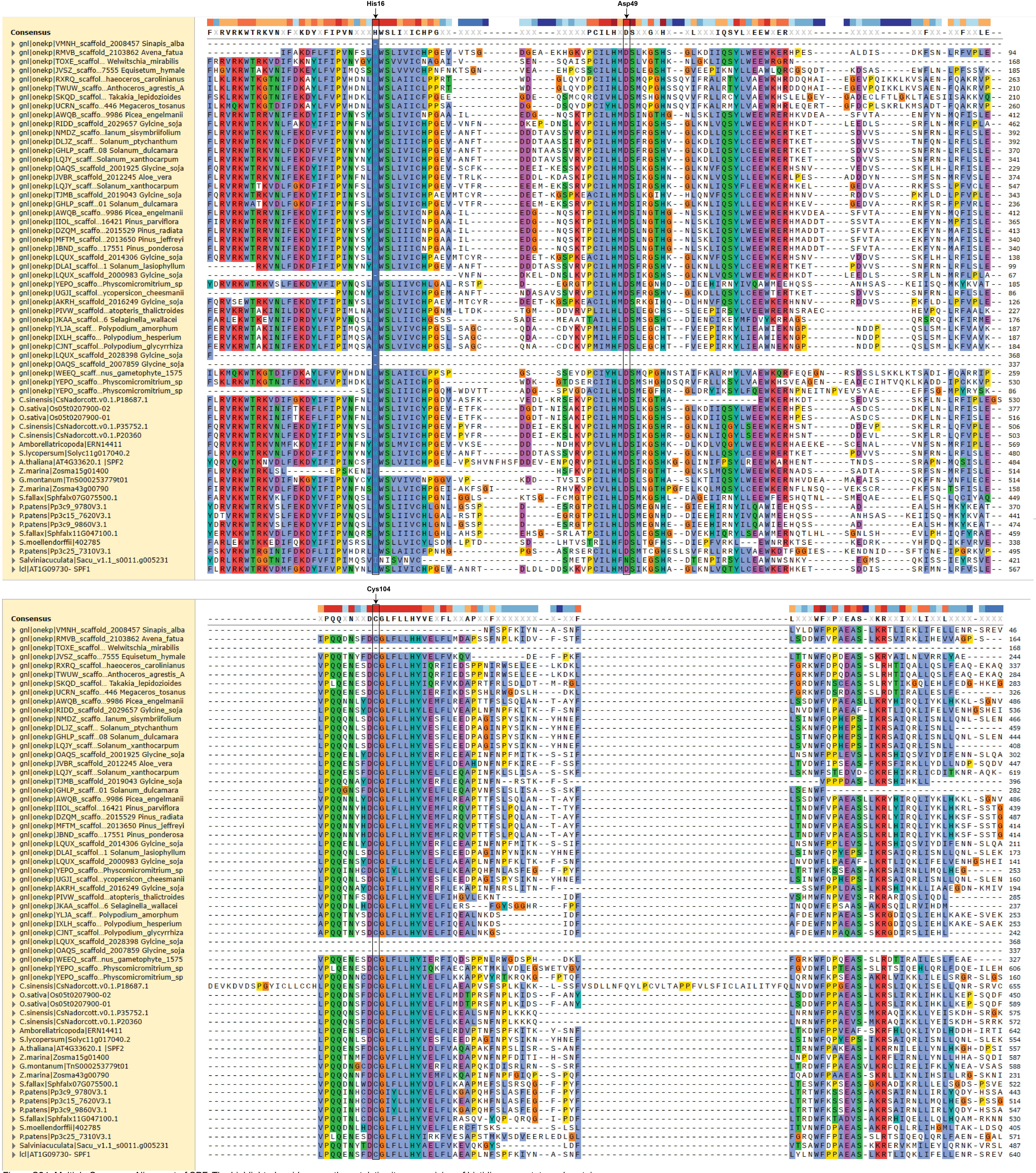

Figure S24: Multiple Sequence Alignment of SPF. The highlighted residues are the catalytic sites comprising of histidine, aspartate and cysteine.

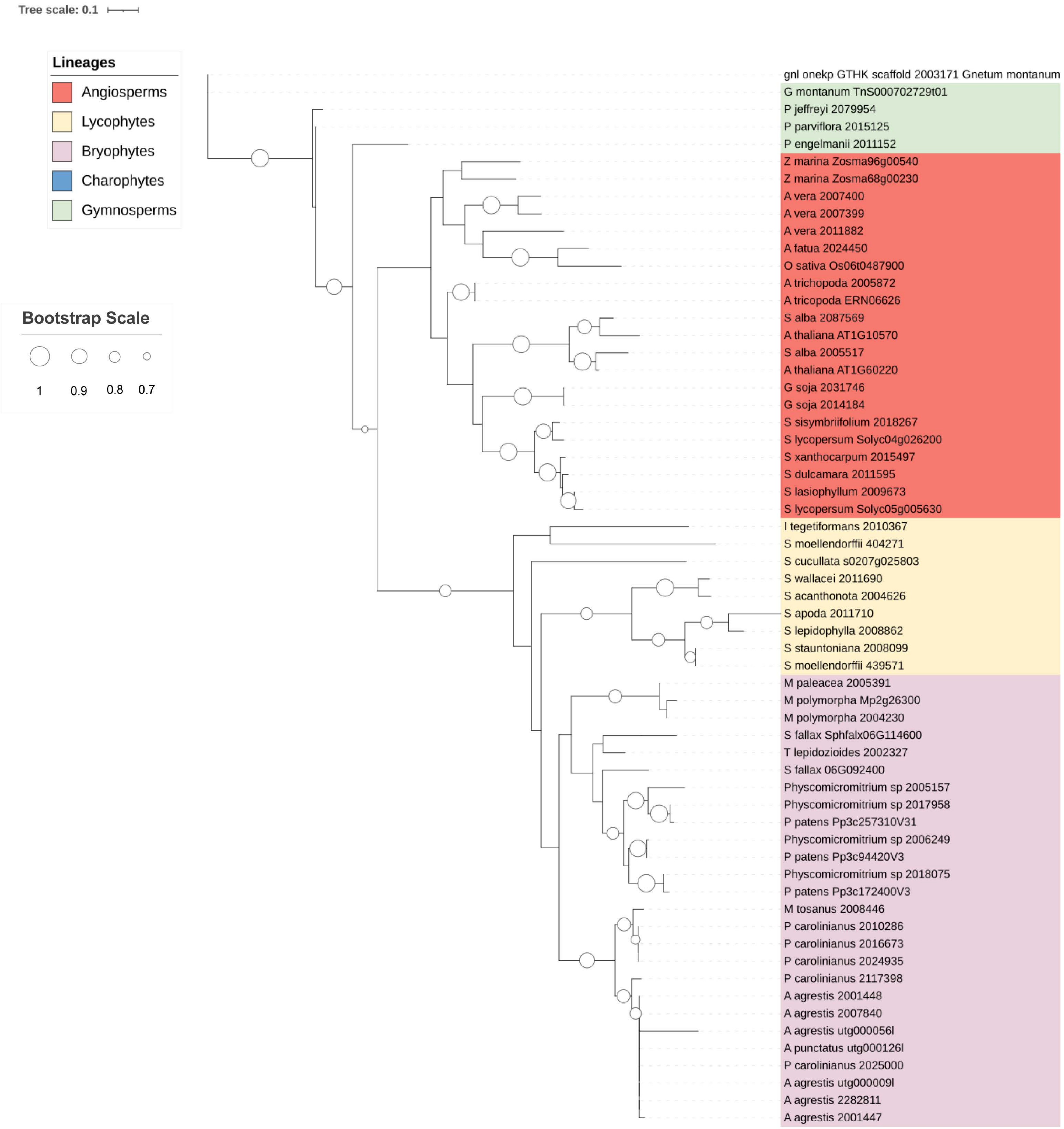

Figure S25: Phylogenetic analysis of SUMO protease OTS (Overly Tolerant to Salt). The corresponding alignments were obtained and processed as indicated in the text and the tree was constructed using PhyML. Bootstrap support was calculated for 1000 trees, and it is indicated in the branches with circles in which the size is proportional to the normalised bootstrap value (between 0 and 1). Only values higher than 0.7 are shown. The tree is drawn to scale, with branch lengths measuring the number of substitutions per site.

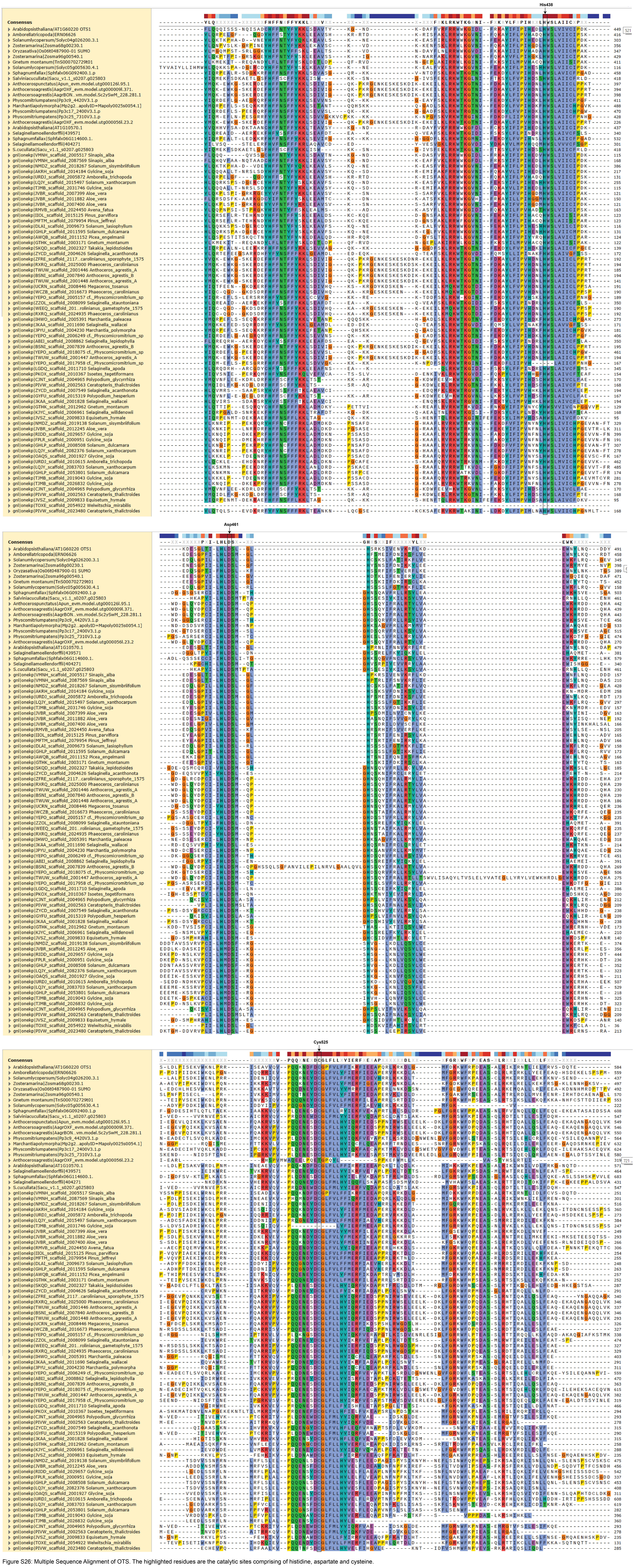

Figure S26: Multiple Sequence Alignment of OTS. The highlighted residues are the catalytic sites comprising of histidine, aspartate and cysteine.

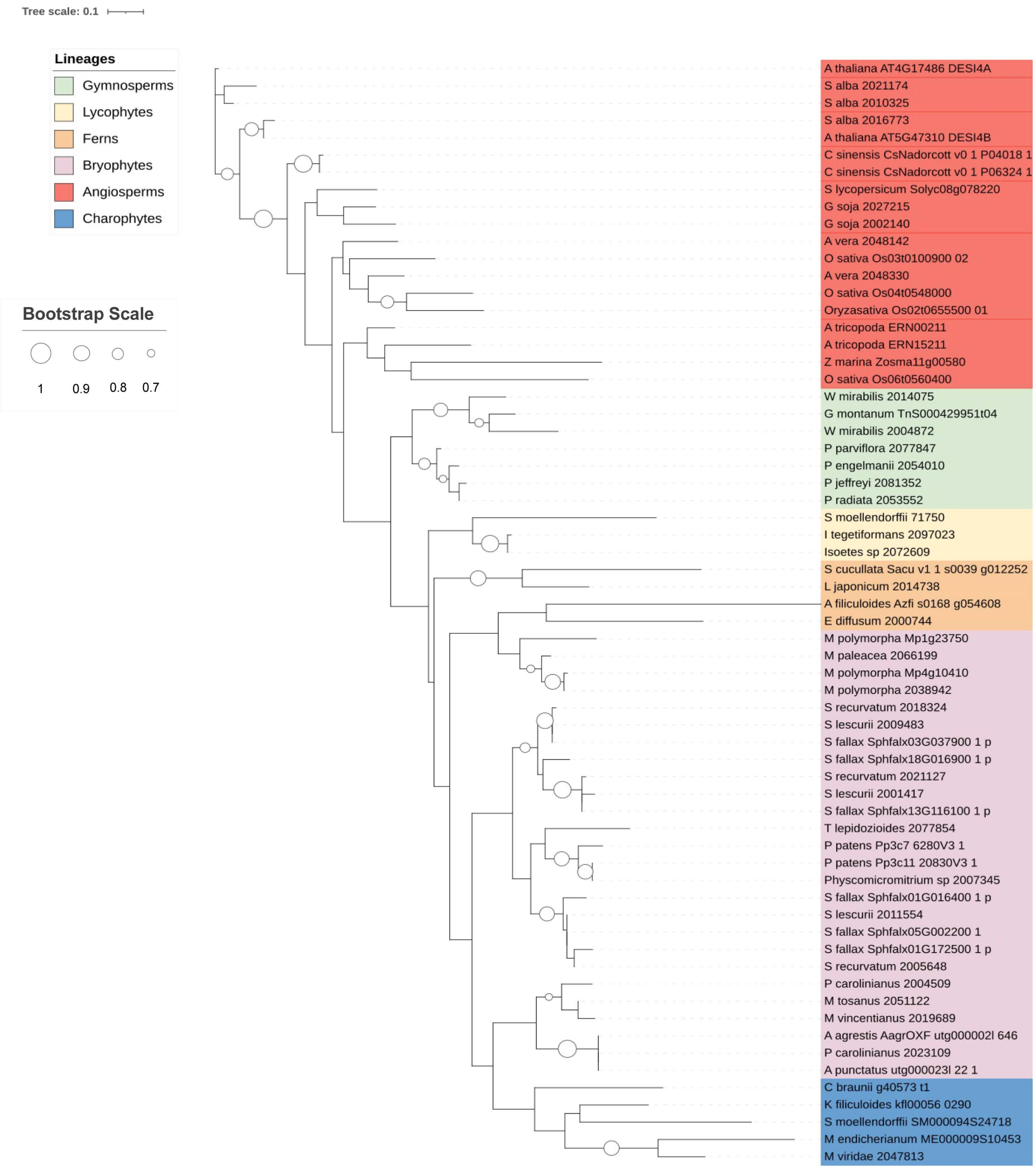

Figure S27: Phylogenetic analysis of SUMO protease DeSI4. The corresponding alignments were obtained and processed as indicated in the text and the tree was constructed using PhyML. Bootstrap support was calculated for 1000 trees, and it is indicated in the branches with circles in which the size is proportional to the normalised bootstrap value (between 0 and 1). Only values higher than 0.7 are shown. The tree is drawn to scale, with branch lengths measuring the number of substitutions per site.
